# Carvacrol Selectively Induces Mitochondria-Related Apoptotic Signaling in Primary Breast Cancer-Associated Fibroblasts

**DOI:** 10.3390/ph19010142

**Published:** 2026-01-14

**Authors:** Nail Besli, Nilufer Ercin, Merve Tokocin, Sümeyra Emine Boluk, Rabia Kalkan Cakmak, Kamil Ozdogan, Talar Vartanoglu Aktokmakyan, Mehtap Toprak, Gulcin Ercan, Merve Beker, Ulkan Celik, Emir Capkinoglu, Yusuf Tutar

**Affiliations:** 1 Department of Medical Biology, Hamidiye School of Medicine, University of Health Sciences Turkey, 34668 Istanbul, Türkiye; 2 Department of General Surgery, University of Health Sciences Istanbul Bagcilar Training and Research Hospital, 34200 Istanbul, Türkiye; 3 Sultan Abdulhamid Han Training and Research Hospital Department of General Surgery, University of Health Sciences, 34668 Istanbul, Türkiye; 4 General Surgery Department, Surp Pirgic Armenian Hospital, 34020 Istanbul, Türkiye; 5 Sultan Abdulhamid Han Training and Research Hospital Department of Pathology, University of Health Sciences, 34668 Istanbul, Türkiye; 6 Department of Medical Biology, Hamidiye International School of Medicine, University of Health Sciences Turkey, 34668 Istanbul, Türkiye; 7 Department of Medical Biology, Institute of Health Sciences, University of Health Sciences Turkey, 34668 Istanbul, Türkiye; 8 Department of General Surgery, School of Medicine, Acibadem Mehmet Ali Aydinlar University, 34638 Istanbul, Türkiye; 9 Division of Biochemistry, Faculty of Pharmacy, University of Health Sciences, 34668 Istanbul, Türkiye; yusuf.tutar@erdogan.edu.tr; 10 Division of Medicinal Biochemistry, Department of Basic Medical Sciences, Faculty of Medicine, Recep Tayyip Erdogan University, 53020 Rize, Türkiye; 11 Training and Research Hospital, Recep Tayyip Erdoğan University, 53020 Rize, Türkiye; 12 Medical Oncology Program, Recep Tayyip Erdogan University, 53100 Rize, Türkiye; 13 Molecular Medicine Program, Recep Tayyip Erdogan University, 53100 Rize, Türkiye

**Keywords:** carvacrol, cancer-associated fibroblasts, breast cancer, apoptosis, NF-κB, PPARα, sirtuins, autophagy

## Abstract

**Background/Objectives**: Cancer-associated fibroblasts (CAFs) are key stromal mediators of breast tumor progression and therapy resistance. Carvacrol, a dietary monoterpenic phenol, exhibits antiproliferative activity in cancer cells, but its effects on primary human breast CAFs remain unclear. This study aimed to determine whether carvacrol selectively induces mitochondria-related apoptotic signaling in breast CAFs while sparing normal fibroblasts (NFs). **Methods**: Primary fibroblast cultures were established from invasive ductal carcinoma tissues (CAFs, *n* = 9) and nonmalignant breast tissues (NFs, *n* = 5) and validated by α-SMA and FAP immunofluorescence. Cells were exposed to 400 μM carvacrol. Apoptosis was assessed by TUNEL assay and BAX/BCL-XL Western blotting. Changes in signaling pathways were evaluated by analyzing PPARα/NF-κB, sirtuin (SIRT1, SIRT3), autophagy-related markers (LAMP2A, p62), and matrix metalloproteinases (MMP-2, MMP-3). In silico molecular docking and 100-ns molecular dynamics simulations were performed to examine interactions between carvacrol and caspase-3 and caspase-9. **Results**: Carvacrol induced a pronounced, time-dependent apoptotic response in CAFs, with TUNEL-based viability declining to approximately 10% of control levels by 12 h and a marked increase in the BAX/BCL-XL ratio. In contrast, NFs exhibited minimal TUNEL positivity and no significant change in BAX/BCL-XL. In CAFs, but not NFs, carvacrol reduced PPARα expression and NF-κB nuclear localization, increased SIRT1 and SIRT3 levels, selectively suppressed MMP-3 while partially normalizing MMP-2, and altered autophagy-related markers (decreased LAMP2A and accumulation of p62), consistent with autophagic stress and possible impairment of autophagic flux. Computational analyses revealed stable carvacrol binding to caspase-3 and caspase-9 with modest stabilization of active-site loops, supporting caspase-dependent, mitochondria-related apoptosis. **Conclusions**: Carvacrol selectively targets breast cancer-associated fibroblasts by inducing mitochondria-related apoptotic signaling while largely sparing normal fibroblasts. This effect is accompanied by coordinated modulation of PPARα/NF-κB, sirtuin, autophagy, and MMP pathways. These findings support further evaluation of carvacrol as a microenvironment-directed adjunct in breast cancer therapy.

## 1. Introduction

Breast cancer most commonly originates from the epithelial cells lining the ducts (~85%) or the lobules (~15%) of the mammary gland. In its earliest stage, neoplastic growth remains confined to the duct or lobule (“in situ”) and is typically paucisymptomatic with a minimal potential for spread; over time, however, invasion across the basement membrane can ensue, followed by regional lymphatic dissemination and ultimately distant metastasis, which accounts for the majority of breast-cancer-related mortality [1]. Contemporary management integrates surgery with radiotherapy and systemic modalities (endocrine therapy, chemotherapy, and targeted biologics), yet therapeutic resistance, relapse, and metastasis remain the principal determinants of poor prognosis [2]. Converging evidence highlights the tumor microenvironment (TME)—and in particular cancer-associated fibroblasts (CAFs)—as key orchestrators of these adverse clinical phenotypes [3,4].

CAFs are the most abundant stromal constituents within the TME and exert pleiotropic, context-dependent effects by remodeling the extracellular matrix (ECM), secreting proteases and cytokines, and dynamically cross-talking with tumor and immune cells [5]. Through ECM reprogramming and matrix stiffening, CAFs can impede immune infiltration and drug penetration while providing a biomechanical and biochemical niche that fosters tumor cell migration, invasion, and other malignant behaviors [3]. Beyond sheer abundance, CAFs display marked heterogeneity in origin, phenotype, and function across breast-cancer subtypes and disease states; multiple markers (e.g., α-SMA, FAP, FSP1/S100A4, PDGFRα/β, NG2, PDPN) capture distinct CAF subsets but none is uniquely specific [6]. Comparative histopathologic studies have shown differential stromal expression of CAF-related proteins between invasive lobular carcinoma (ILC) and invasive ductal carcinoma (IDC), as well as stromal-type-dependent variation in ductal carcinoma in situ (DCIS) [7,8]. Importantly, transcriptomic profiling indicates that stromal fibroblasts isolated from invasive breast tumors adopt gene-expression programs distinct from normal breast fibroblasts, underscoring a stromal—rather than epithelial—determinant in disease progression [9].

Matrix metalloproteinases (MMPs), notably MMP-1, MMP-2, MMP-3, and MMP-9, are frequently elevated in aggressive breast tumors and promote invasion and angiogenesis; their transcription is driven in part by AP-1 and Nuclear factor-κB (NF-κB), two inflammation-responsive transcriptional axes that integrate cytokine, growth factor, and stress cues [10,11,12]. NF-κB is a crucial link between chronic inflammation and cancer development, driving processes such as cell proliferation, blocking programmed cell death, promoting the formation of new blood vessels, and supporting both epithelial–mesenchymal transition and metastasis [13]. By understanding its role, we can grasp the significant impact of chronic inflammation on oncogenesis. In line with this, paracrine IL-6 signaling from CAFs to DCIS cells can accelerate the transition from pre-invasive to invasive phenotypes, with reduced IκBα and activation of NF-κB/AP-1 pathways observed in relevant models [14].

Given the centrality of stromal programs in breast cancer, there is growing interest in natural small molecules that modulate pro-tumorigenic TME pathways. Carvacrol (CV)—a monoterpenic phenol (2-methyl-5-isopropylphenol) abundant in thyme and related aromatic plants—has shown anticancer activity across several carcinoma models, where it triggers apoptosis, enforces cell-cycle arrest, attenuates metastatic traits, and targets MAPK, Notch, PI3K–AKT–mTOR and related cascades [15]. In breast cancer lines, CV has been reported to down-regulate Bcl-2, up-regulate Bax, and induce caspase-3/-6/-9 activation in a dose-dependent manner, consistent with mitochondrial apoptosis [16,17]. Collectively, in vitro and in vivo studies support the notion that CV is a bioactive compound with potential anti-cancer properties, though defining effective doses, toxicity windows, and precise mechanisms—particularly within patient-derived stromal contexts—requires more rigorous, multi-scale investigation [15].

Autophagy, including macroautophagy and chaperone-mediated autophagy (CMA), is increasingly recognized as a context-dependent regulator of breast cancer progression and stromal fitness, particularly through its role in cancer-associated fibroblast (CAF) activation and tumor–stroma crosstalk. In CAFs and the broader breast tumor microenvironment, perturbations of LAMP2A-dependent CMA and p62/SQSTM1 signaling, together with stress-responsive sirtuins (e.g., SIRT1/SIRT3), have been linked to proteostasis control, metabolic adaptation and therapy resistance, positioning these pathways as attractive yet still underexplored targets for stromal-directed intervention [18,19,20,21,22].

Building on these gaps, we use patient-derived, stromal CAF cultures (not epithelial lines) alongside normal fibroblasts to test whether CV reprograms TME-linked signaling rather than merely intoxicating tumor epithelium. We hypothesize that CV blunts NF-κB nuclear translocation with concordant down-modulation of MMP-1/MMP-3, drives mitochondria-related apoptotic signaling (TUNEL; ↑BAX/BCL-XL), and remodels metabolic stress nodes—PPARα/PPARγ and SIRT1/SIRT3—while imposing proteostasis stress indexed by autophagy/CMA markers (p62/SQSTM1 accumulation with LAMP2A loss); these endpoints are quantified by Western blot and subcellular localization (immunofluorescence). Our prior work and others’ show that pharmacologic NF-κB inhibition (e.g., metformin) suppresses MMP-2/MMP-9 and invasion in breast cancer systems, underscoring the tractability of this node [23,24]. Altogether, this framework positions carvacrol as a stroma-centric, multi-target candidate capable of functionally reprogramming CAFs and attenuating malignant progression in breast cancer.

## 2. Results

### 2.1. In Silico Calculations

**Figure 1:**
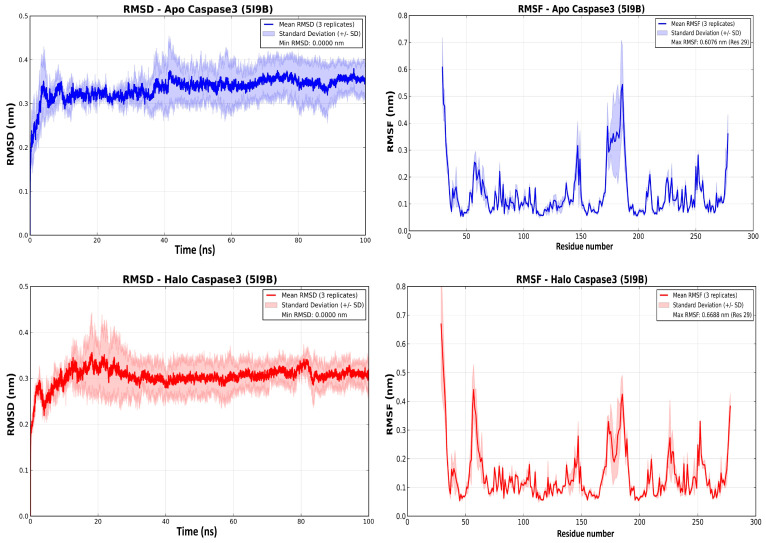
MD stability and flexibility of caspase-3 (PDB 5I9B) with and without carvacrol. (**Top-left**): Apo RMSD (nm) vs. time (ns). (**Bottom-left**): Holo (carvacrol-bound) RMSD. (**Top-right**): Apo RMSF (nm) per residue. (**Bottom-right**): Holo RMSF. Curves show mean of three independent 100-ns replicates; shading denotes ± SD. Simulations used SPC water, OPLS-AA force field, 300 K/1 bar, with standard EM, NVT, and NPT equilibration.

**Figure 2:**
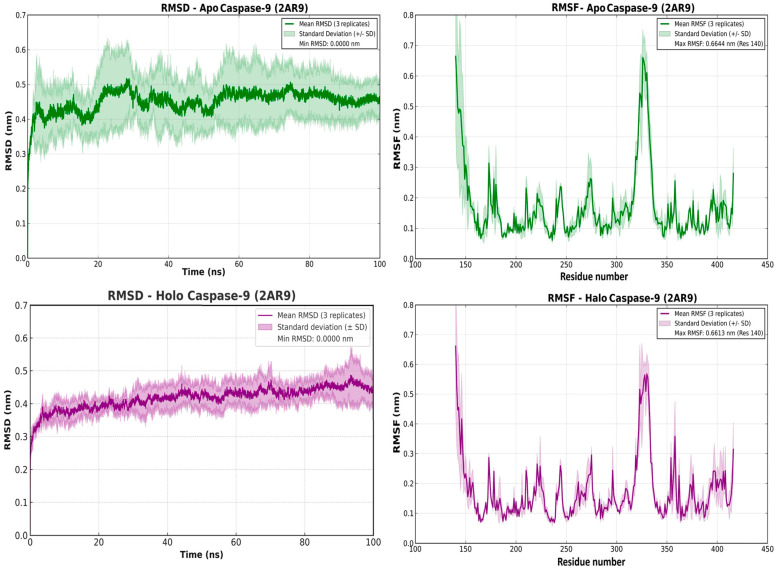
MD stability and flexibility of caspase-9 (PDB 2AR9) with and without carvacrol. Panels as in Figure 2: apo (**top**) and holo (**bottom**) RMSD (**left**) and RMSF (**right**), mean of triplicates ± SD across 100 ns. Holo trajectories show markedly reduced global drift and localized flexibility suppression around the ~330–350 region. Full MD parameters are described in Methods.

Blind docking of carvacrol identified plausible pockets on caspase-3 and caspase-9. For caspase-3 (PDB 5I9B), the best Vina score was −5.4 kcal·mol^−1^, with H-bonds to Gln225 and Tyr226 and auxiliary hydrophobic contacts (Arg238/Lys242). For caspase-9 (PDB 2AR9), the top pose scored −5.2 kcal·mol^−1^ and was purely hydrophobic/π–alkyl (Ile154, Phe412, aliphatic Lys414), with no hydrogen bonds observed. Annotated interaction maps and 3D poses are shown in Supplementary File S2 (Figure 1 and Figure 2).

**Figure 3:**
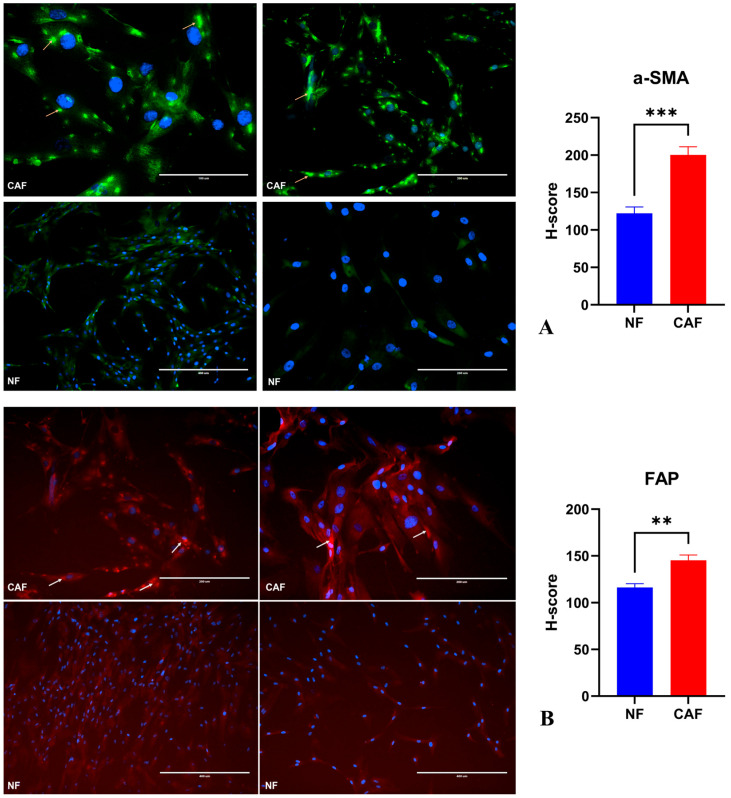
Immunofluorescent characterization of CAFs versus normal fibroblasts. (**A**) Representative images of α-SMA (GFP, green) with DAPI (blue) in CAF and NF cultures; right, semi-quantitative H-score analysis showing increased α-SMA signal in CAFs (*** *p* < 0.001). (**B**) Representative images of FAP (Texas-red) with DAPI (blue); right, H-score analysis indicating higher FAP levels in CAFs relative to NFs (** *p* < 0.01). Arrows mark positive cells. Images were acquired at 20× from five randomly selected fields per well; *n* = 3 independent experiments (biological replicates), data are mean ± SD. Scale bars: 200 μm.

Molecular dynamics (100 ns; 3 replicates) supported stable engagement of carvacrol with both caspases (Figure 1 and Figure 2). For caspase-3 (PDB 5I9B), the apo backbone RMSD drifted to ~0.33–0.38 nm, whereas the carvacrol-bound (“holo”) complex rapidly plateaued at ~0.28–0.32 nm, with dampened RMSF along flexible loops (~res. 45–60 and 175–205) (Figure 2). For caspase-9 (PDB 2AR9), the apo state fluctuated around ~0.45–0.50 nm, while the CV-bound holo trajectories exhibited minimal backbone deviation (~0.015–0.020 nm across three 100-ns runs) and reduced RMSF in the mobile loop region (~residues 330–350) (Figure 3). Together with docking affinities (caspase-3: −5.4 kcal/mol; caspase-9: −5.2 kcal/mol) and hydrophobic-only contacts in the 2AR9 pose (no hydrogen bonds observed), these simulations indicate that carvacrol favors a more compact, dynamically restrained enzyme state (see Supplementary File S2, Figures S1 and S2 for representative poses).

Docking and triplicate 100-ns MD indicate that carvacrol forms stable, predominantly hydrophobic complexes with caspase-3 and caspase-9 and dampens loop dynamics, consistent with conformational stabilization; together with increased TUNEL indices and an elevated BAX/BCL-XL ratio, these data support CV-driven pro-apoptotic signaling in CAFs, while the direction of direct caspase modulation (activation vs. inhibition) awaits enzymatic validation.

### 2.2. Primary Breast CAF Characterization

To verify stromal identity, we profiled canonical CAF markers by immunofluorescence. Compared with patient-matched normal fibroblasts (NF), CAF cultures exhibited robust up-regulation of α-SMA and FAP, with intense cytoplasmic/filamentous staining and increased H-scores (two-tailed tests with multiple-comparison correction; α-SMA: *** *p* < 0.001; FAP: ** *p* < 0.01). These data confirm a myofibroblast-like, tumor-stroma phenotype in the CAF preparations (Figure 3).

All tumors from which CAF and NF cultures were derived were invasive ductal carcinomas. Among the nine breast cancer cases, eight were histological grade II and one was grade III. Estrogen receptor (ER) and progesterone receptor (PR) expression was positive in eight of nine tumors, consistent with a predominantly luminal-like phenotype; one case was ER/PR-negative. Ki-67 labeling indices ranged from approximately 5% to 45%. Detailed clinicopathological variables, including ER/PR status, histological grade, Ki-67 and availability of matched adjacent normal tissue, are summarized in Supplementary File S1 Table S1.

### 2.3. Dose–Response and Working Concentration Selection by MTT Assay

**Figure 4:**
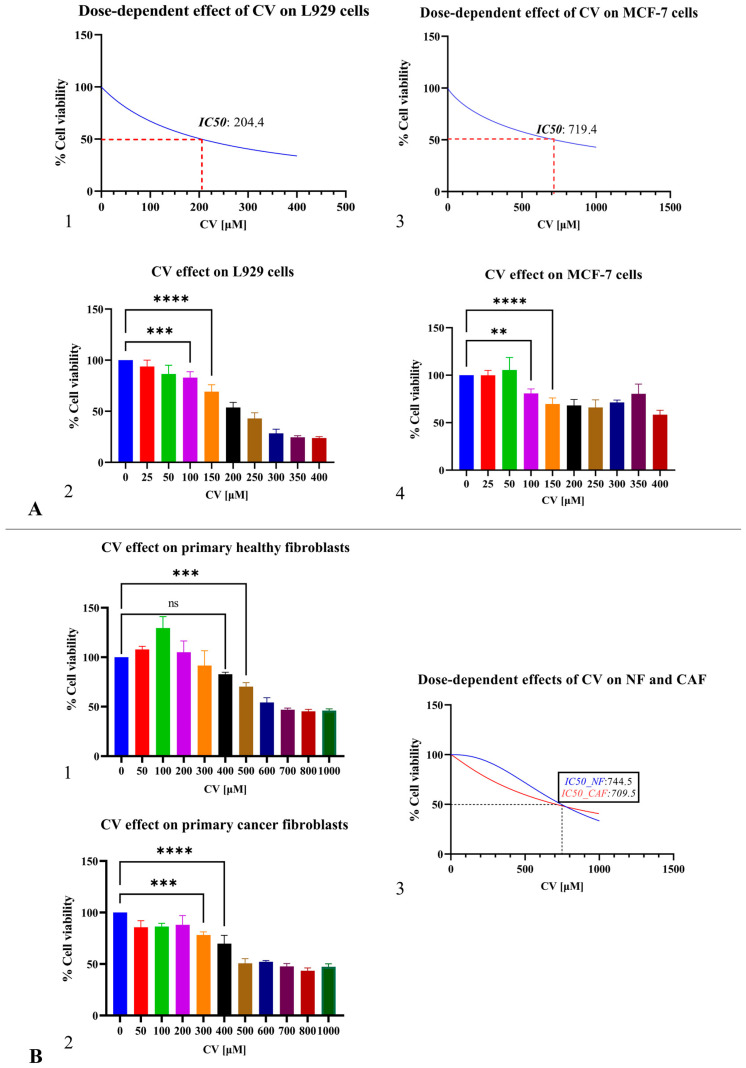
(**A**) Cell-line panel. Left: L929 viability curve with IC_50_ ≈ 204 μM; bar plot shows mean ± SD (*n* = 3) across 0–400 μM with one-way ANOVA. Right: MCF-7 curve with IC_50_ ≈ 719 μM; bar plot as above demonstrating significant loss at ≤400 μM. (**B**) Primary stromal panel. Left: NF dose series (0–1000 μM); IC_50_ ≈ 745 μM. Middle: CAF dose series (0–1000 μM); IC_50_ ≈ 709 μM. Right: overlay of NF (blue) and CAF (red) sigmoids wFor all downstream assays (WB, IF, TUNEL), ith dotted line at 50% viability and vertical marker at 400 μM (sub-IC_50_ for both). Significance: ns not significant, ** *p* < 0.01, *** *p* < 0.001, **** *p* < 0.0001; one-way ANOVA with multiple-comparison test; *n* = 3 independent repeats.

Representative pre-treatment images of L929 and MCF-7 cultures are shown in Supplementary File S1 (Figures S3 and S4). Carvacrol (CV) produced a clear, dose-dependent loss of viability in L929 and MCF-7 (Figure 4(A1–A4)). To define non-toxic working ranges, IC_50_ values were calculated (Figure 4(A1–A3)). One-way ANOVA with Tukey’s multiple comparisons confirmed significant differences across concentrations in L929 (Figure 4(A2); pairwise *** *p* < 0.0005, **** *p* < 0.0001) and in MCF-7 (Figure 4(A4); pairwise *** *p* = 0.0044 to **** *p* < 0.0001).

Primary stromal fibroblasts (0–1500 μM, 24 h). The same dose series applied to normal fibroblasts (NF) and cancer-associated fibroblasts (CAF) also yielded concentration-dependent declines (Figure 4(B1–B3)). ANOVA/Tukey’s indicated a significant trend in NF (Figure 4(B1); *** *p* = 0.0004) and robust effects in CAF (Figure 4(B2); *** *p* < 0.001, **** *p* < 0.0001). Nonlinear fits provided IC_50_ estimates summarized in Figure 4(B3).

For all downstream assays (WB, IF, TUNEL), we selected 400 μM CV for 24 h as the working concentration because this condition is below the IC_50_ values for both NF and CAF, causes only a modest, non-significant reduction in NF viability at 400 μM (Figure 4(B1)), yet significantly reduces CAF viability (Figure 4(B2)) and exerts a measurable cytostatic/cytotoxic effect in MCF-7 cells (Figure 4(A4)). This sub-IC_50_, non-saturating setting preserves adequate cell numbers while enabling biologically informative comparisons across groups.

### 2.4. Carvacrol Induces Time-Dependent Apoptosis in CAFs

**Figure 5:**
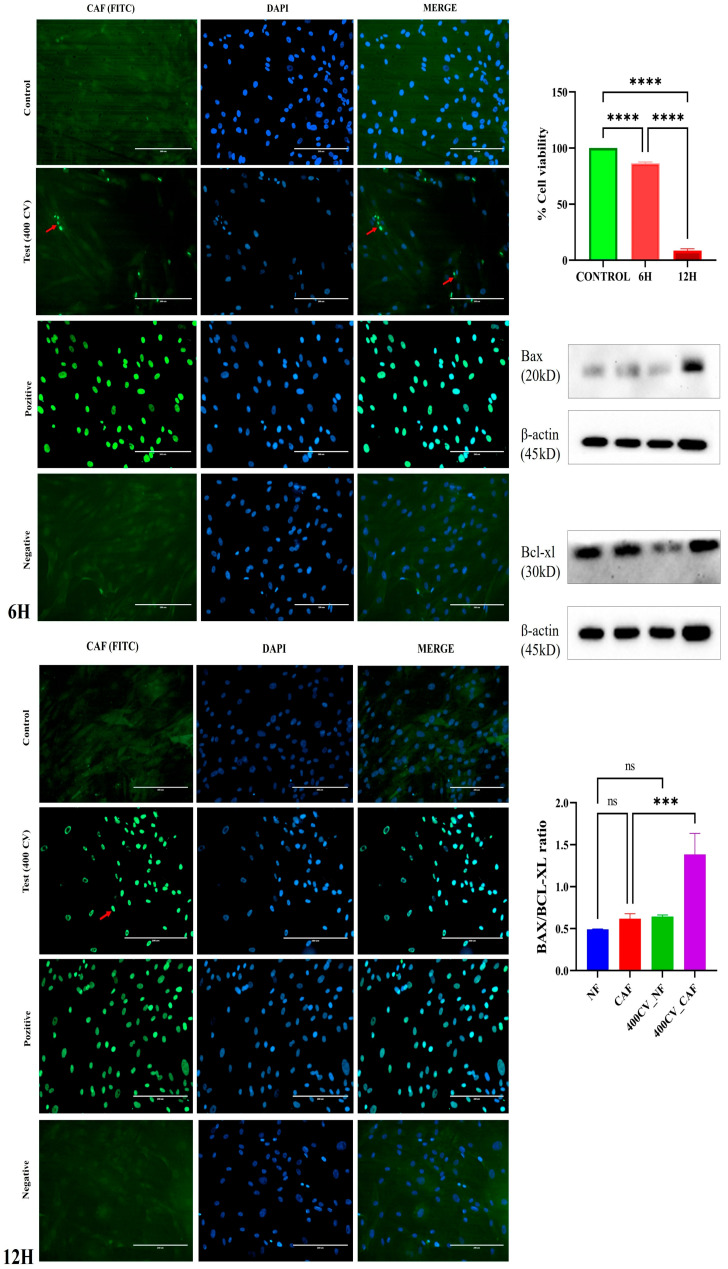
Carvacrol triggers apoptosis in CAFs: TUNEL survival readout and BAX/BCL-XL shift. Representative TUNEL IF images of CAFs at Control, 6 h (400 μM CV), and 12 h (400 μM CV) are shown (FITC = TUNEL, DAPI = nuclei; scale bars = 200 μm). Bars quantify % Surviving Cells = 100 × (TUNEL-negative nuclei/total DAPI nuclei) from ≥5 random fields per replicate (*n* = 3). Statistics: one-way ANOVA with Tukey (**** *p* < 0.0001). Right: Western blots for BAX and BCL-XL with β-actin loading control and the BAX/BCL-XL ratio (mean ± SD, *n* = 3); one-way ANOVA/Tukey (*** *p* < 0.001; ns, not significant). Positive and negative assay controls are included. Red arrows indicate representative FITC-positive cells exhibiting apoptotic nuclear morphology (condensed/fragmented nuclei in DAPI), as confirmed in the merged images.

**Figure 6:**
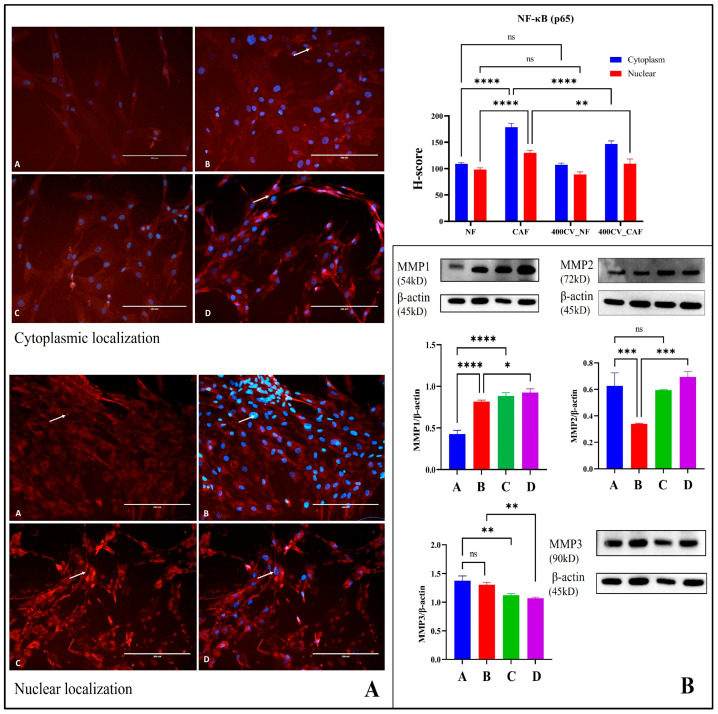
Cellular (cytoplasmic and nuclear) localization of NF-κB (p65) by immunofluorescence and Western blot analysis of MMPs in NF and CAF with or without carvacrol. (**A**) Representative IF images (p65 texas red, DAPI blue) for (A) NF, (B) CAF, (C) 400 CV_NF (NF + 400 μM CV, 24 h), (D) 400 CV_CAF (CAF + 400 μM CV, 24 h) at 20× (EVOS^®^ FL Auto). Scale bar: 200 μm. Semi-quantitative H-score analysis of p65 localization. H-scores (mean ± SEM, *n* = 3) for cytoplasmic (blue bars) and nuclear (red bars). (**B**) MMPs representative blots (upper panels) with β-actin loading control and corresponding densitometry (mean ± SEM, *n* = 3). White arrows indicate representative cells showing enhanced immunofluorescence signal and/or subcellular localization of the indicated proteins. Statistical significance is denoted as follows: ns, not significant; * *p* < 0.05; ** *p* < 0.01; *** *p* < 0.001; **** *p* < 0.0001.

Consistent with the TUNEL-derived survival index, 400 μM carvacrol (CV) progressively shifted the mitochondrial apoptotic balance in CAFs. The BAX/BCL-XL ratio showed a modest, non-significant increase at 6 h relative to untreated CAFs, but rose robustly by 12 h (one-way ANOVA with Tukey; **** *p* < 0.001 vs. control and vs. 6 h), indicating a pro-apoptotic tilt at later time points (Figure 5, right). In matched NF cultures, CV produced no significant change in BAX/BCL-XL across time (ns), in line with the minimal TUNEL positivity observed in NFs (Supplementary File S2, Figure S3). Together with the decline in the TUNEL-derived survival index (Figure 6, left), these data indicate that CV induces time-dependent apoptotic commitment in CAFs while sparing normal fibroblasts at the tested dose. In Figure 5, TUNEL data are expressed as a survival index (100 × [1 − TUNEL^+^/total nuclei]) rather than % TUNEL-positive cells, to facilitate comparison with other viability readouts.

### 2.5. NF-κB/MMP Axis Modulation by Carvacrol in Breast-Tumor Stroma

Immunofluorescence (IF) demonstrated higher cytoplasmic and nuclear p65 signals in CAF vs. NF (both **** *p* < 0.0001), indicative of pathway activation. Treatment with 400 μM CV reduced nuclear H-scores in CAF compared with untreated CAF (** *p* < 0.01) and shifted p65 toward the cytoplasm, while NF showed no significant change (ns) (see Figure 6A). Together with the MMP data, these findings support a model in which CV dampens NF-κB signaling in CAFs, selectively impacting stroma-driven inflammatory/ECM programs.

As expected, baseline MMP-1 was higher in CAF than NF (NF vs. CAF, **** *p* < 0.0001). Following CV, MMP-1 did not decrease; instead, expression in both NF and CAF rose toward or above the control CAF level (NF vs. 400 CV_NF, **** *p* < 0.0001; CAF vs. 400 CV_CAF, *p* < 0.05; 400 CV_NF vs. 400 CV_CAF, ns) (see Figure 6B). Thus, unlike MMP-3, MMP-1 is not repressed by CV and may be induced in NF, suggesting context-dependent remodeling or inflammatory signaling that warrants mechanistic follow-up. Densitometry revealed lower MMP-2/β-actin in CAF vs. NF (* *p* < 0.001). CV did not change MMP-2 in NF (ns) but significantly increased MMP-2 in CAF, restoring it toward the NF range (CAF vs. 400 CV_CAF, *** *p* < 0.001). This pattern suggests that CV partially normalizes MMP-2 levels in CAFs, consistent with modulation of extracellular-matrix turnover (see Figure 6B, right panels). As for MMP-3, Western blotting showed no baseline difference in MMP-3 between normal fibroblasts (NF) and cancer-associated fibroblasts (CAF) (NF vs. CAF, ns). In contrast, 400 μM carvacrol (CV, 24 h) significantly reduced MMP-3 in both lineages (NF vs. 400 CV_NF, ** *p* < 0.01; CAF vs. 400 CV_CAF, ** *p* < 0.01 (see Figure 6B at bottom). These data indicate a broad MMP-3-lowering effect of CV that is independent of fibroblast subtype, consistent with an anti-invasive, matrix-modulating action.

Overall, CV exerts a stroma-selective dampening of NF-κB in CAFs—evidenced by reduced p65 nuclear localization—while reprogramming ECM remodeling in a nonuniform manner, lowering MMP-3 and restoring MMP-2 toward NF baselines but not suppressing (and in NF modestly inducing) MMP-1, consistent with context-dependent normalization of CAF programs rather than global MMP blockade.

### 2.6. Carvacrol Selectively Down-Modulates PPARα in CAFs While Sparing PPARγ

**Figure 7:**
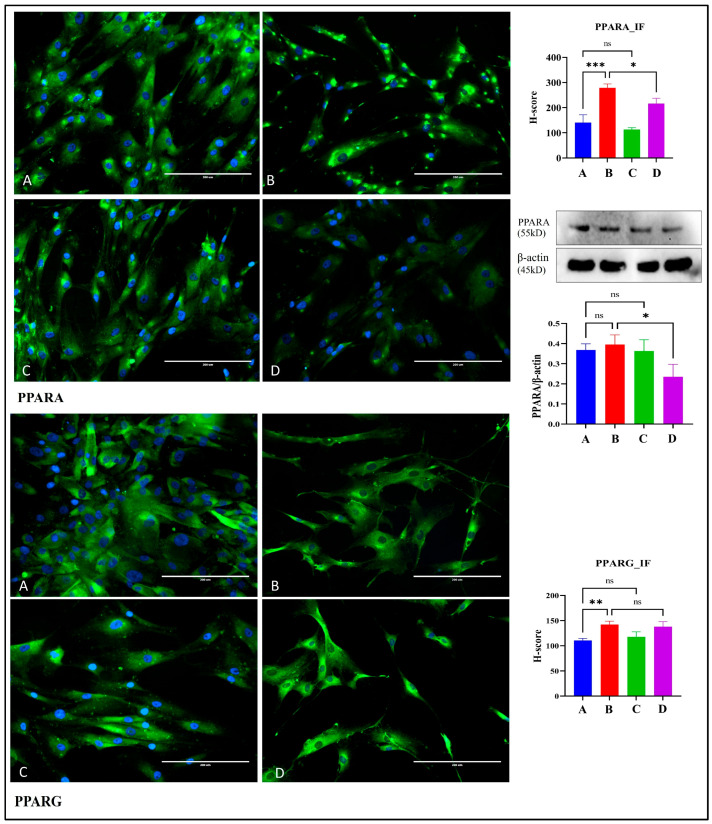
CV remodels PPAR signaling in fibroblasts (IF and WB). Representative IF micrographs (GFP/green for target; DAPI/blue for nuclei) and H-score bar plots for PPARA (top block) and PPARG (bottom) in A: NF, B: CAF, C: NF + CV (400 μM, 24 h), D: CAF + CV (400 μM, 24 h). Scale bars: 200 μm. Right-side graphs summarize H-scores (mean ± SEM, *n* = 3). Middle-right panels show representative immunoblots with β-actin loading control and corresponding densitometry (PPARA/β-actin; mean ± SEM, *n* = 3). Statistical significance is denoted as follows: ns, not significant; * *p* < 0.05; ** *p* < 0.01; *** *p* < 0.001.

Immunofluorescence (IF) H-score analyses showed higher PPARA signal in CAF than NF (*** *p* < 0.001). Exposure to 400 μM carvacrol (CV, 24 h) reduced PPARA staining in CAF relative to untreated CAF (* *p* < 0.05), whereas NF showed no change with CV (ns). By Western blot, PPARA/β-actin at baseline was similar in NF and CAF (ns); CV again produced no effect in NF (ns) but decreased PPARA in CAF compared with CAF control and NF + CV (* *p* < 0.05). For PPARG, IF confirmed a higher basal signal in CAF than NF (** *p* < 0.01), yet CV did not significantly alter PPARG in either lineage (ns). Collectively, these data indicate that CV preferentially down-regulates PPARα in activated stromal fibroblasts, while PPARγ remains largely unchanged, consistent with a CAF-biased modulation of PPAR signaling rather than a global PPAR suppression (Figure 7).

### 2.7. Autophagy/CMA (Chaperone-Mediated Autophagy) and Sirtuin Signaling

**Figure 8:**
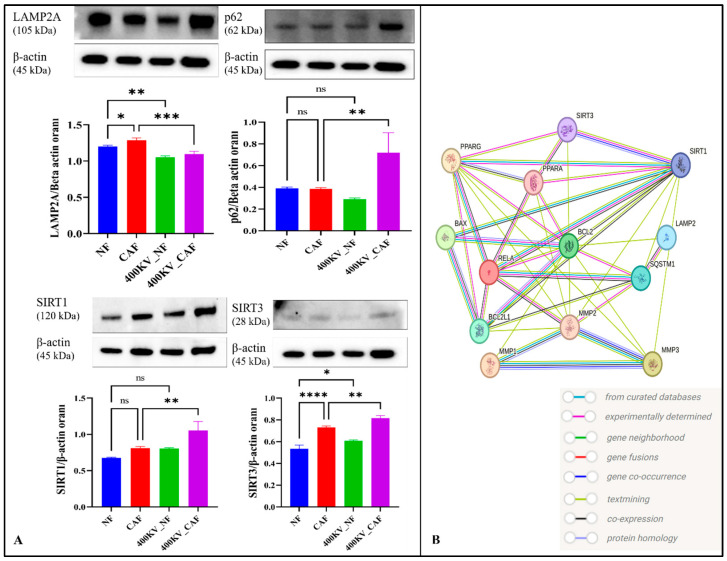
Carvacrol impairs chaperone-mediated autophagy and activates SIRT1/SIRT3 signaling in CAFs. (**A**) Representative Western blots (**top**) and densitometric quantification (**bottom**; mean ± SEM, *n* = 3) of LAMP2A, p62/SQSTM1, SIRT1 and SIRT3 in four groups: NF (normal fibroblasts), CAF (cancer-associated fibroblasts), NF + CV (400 μM, 24 h) and CAF + CV (400 μM, 24 h). Band intensities are normalized to β-actin. Statistical significance is denoted as follows: ns, not significant; * *p* < 0.05; ** *p* < 0.01; *** *p* < 0.001; **** *p* < 0.0001. (**B**) STRING protein–protein interaction network for the CV-responsive proteins LAMP2A, SQSTM1/p62, SIRT1, SIRT3, BAX, BCL2L1, MMP1, MMP2, MMP3, PPARG, PPARA and RELA. Nodes represent proteins and edges indicate known or predicted functional associations. Edge colors correspond to different evidence channels (from curated databases, experimentally determined interactions, gene neighborhood, gene fusions, gene co-occurrence, text-mining, co-expression and protein homology), as indicated in the STRING legend.

Carvacrol altered autophagy-associated markers and sirtuin levels in a CAF-selective manner (Figure 8A). At baseline, LAMP2A (CMA receptor) was higher in CAF vs. NF (* *p* < 0.05). CV (400 μM, 24 h) reduced LAMP2A in both lineages, with a stronger decrement in NF (*p* < 0.01–*p* < 0.001). p62/SQSTM1 did not differ between NF and CAF at baseline (ns), but accumulated in CAF after CV (** *p* <0.01) while showing a slight reduction in NF (ns), p62/SQSTM1 accumulated in CAF after CV, a pattern compatible with reduced autophagic clearance/CMA capacity in CV-treated CAFs, although direct flux measurements were not performed. SIRT1 was similar at baseline (ns) and rose only in CAF after CV (** *p* < 0.01), whereas SIRT3 was intrinsically higher in CAF (**** *p* < 0.0001) and increased further with CV, most prominently in CAF (*p* < 0.05–0.01). Considering the apoptosis readouts (BAX/BCL-XL ratio and TUNEL; see Figure 5), these data suggest that CV modulates autophagy/CMA markers and engages SIRT1/SIRT3-linked stress programs in CAFs, biasing the stroma toward pro-apoptotic outcomes while sparing NF.

## 3. Discussion

The present investigation delineates a novel paradigm wherein carvacrol (CV), a monoterpenic phenol abundant in Lamiaceae species, selectively orchestrates mitochondria-dependent apoptotic cascades in primary breast cancer-associated fibroblasts (CAFs) while preserving the viability of patient-matched normal fibroblasts (NFs). This stromal selectivity, substantiated by TUNEL-based survival assays, modulation of the BAX/BCL-XL ratio, and complementary in silico molecular dynamics simulations, underscores the translational potential of CV as a microenvironment-directed adjunct in breast oncology. Herein, we contextualize our findings within the contemporary understanding of CAF biology, metabolic reprogramming, and autophagy-dependent proteostasis, while delineating mechanistic convergence points warranting further interrogation.

Cancer-associated fibroblasts constitute the predominant cellular constituent of the tumor microenvironment (TME) and exert pleiotropic, context-dependent effects that facilitate neoplastic progression, therapeutic resistance, and metastatic dissemination [25]. Conventional chemotherapeutic paradigms predominantly target rapidly proliferating epithelial malignancies, inadvertently sparing stromal compartments that subsequently orchestrate relapse and treatment failure. Our demonstration that 400 μM CV induces time-dependent apoptosis in CAFs—evidenced by progressive TUNEL positivity (declining to ~10% viability at 12 h) and a robust elevation in the BAX/BCL-XL ratio—while causing only modest, non-significant viability loss in NFs under the same sub-IC_50_ conditions, represents a conceptual departure from epithelium-centric therapeutic strategies. This differential susceptibility may reflect intrinsic metabolic vulnerabilities and redox imbalances characteristic of activated stromal fibroblasts, which exhibit heightened oxidative phosphorylation, glycolytic flux, and mitochondrial stress relative to quiescent NFs [26,27].

The BAX/BCL-XL rheostat serves as a critical determinant of mitochondrial outer membrane permeabilization (MOMP) and subsequent caspase activation. Our Western blot analyses revealed a statistically significant increase in the BAX/BCL-XL ratio in CV-treated CAFs at 12 h (*p* < 0.001), consistent with a pro-apoptotic shift in BCL-2 family protein equilibrium. This observation aligns with recent experimental evidence demonstrating that carvacrol induces concentration-dependent apoptosis in breast cancer cells via upregulation of pro-apoptotic BAX and downregulation of anti-apoptotic BCL-2, with IC50 values ranging from 100–320 μM across multiple breast cancer cell lines, including MCF-7, MDA-MB-231, and HCC1937 [28]. Mechanistically, carvacrol-mediated apoptosis in MCF-7 cells has been linked to suppression of the PI3K/AKT survival pathway, which converges with BAX/BCL-2 modulation to amplify mitochondrial apoptotic signaling and induce G0/G1 cell cycle arrest [29]. Notably, the absence of BAX/BCL-XL modulation in NFs suggests that CV-induced apoptosis is contingent upon CAF-specific signaling aberrations, potentially including constitutive NF-κB activation, dysregulated PPAR signaling, or impaired autophagic flux—each of which was interrogated in our experimental framework.

To elucidate the molecular underpinnings of CV-mediated apoptosis, we employed blind docking and triplicate 100-ns molecular dynamics (MD) simulations targeting caspase-3 (PDB 5I9B) and caspase-9 (PDB 2AR9). Caspase-3, an executioner protease, and caspase-9, an initiator caspase integral to the intrinsic apoptotic pathway, represent canonical effectors of mitochondria-dependent cell death. Our docking analyses yielded binding affinities of −5.4 kcal/mol (caspase-3) and −5.2 kcal/mol (caspase-9), with CV engaging the active-site cleft via hydrogen bonding (Gln225, Tyr226 in caspase-3) and hydrophobic/π-alkyl interactions (Ile154, Phe412, Lys414 in caspase-9). Subsequent MD trajectories revealed that CV stabilizes both caspases, as evidenced by reduced backbone root-mean-square deviation (RMSD) and dampened root-mean-square fluctuation (RMSF) in flexible loop regions. Specifically, the holo (CV-bound) caspase-3 complex exhibited RMSD values of ~0.28–0.32 nm versus ~0.33–0.38 nm for the apo state, with analogous stabilization observed for caspase-9, where the apo form gradually increased and plateaued around ~0.45–0.50 nm, whereas the CV-bound complex converged to a slightly lower and more compact plateau of approximately 0.43 ± 0.05 nm (0.38–0.48 nm across three 100-ns replicates; Figure 2). These computational findings, obtained from triplicate 100-ns MD trajectories, indicate that CV can act as a conformational stabilizer of caspase-3 and caspase-9 active-site loops, narrowing backbone RMSD and RMSF distributions relative to the apo state (Figure 1 and Figure 2). When considered together with the robust increase in TUNEL positivity and the BAX/BCL-XL shift in CAFs, these results strongly support the involvement of caspase-dependent mitochondrial apoptosis, although direct enzymatic measurements of caspase activity and PARP cleavage will be required to definitively confirm this mechanism. Experimental validation of caspase engagement has been reported in multiple cancer cell types, wherein carvacrol treatment triggers cytochrome c release from mitochondria, subsequent activation of caspase-9 (initiator) and caspase-3 (executioner), and PARP cleavage—hallmarks of intrinsic apoptotic pathway activation [30]. However, the directionality of this modulation—whether CV acts as a direct activator or stabilizes an active conformation—remains to be elucidated through enzymatic kinetic assays. Precedent exists for natural compounds exerting dual effects on caspases; for instance, resveratrol has been shown to enhance caspase-3 activity in specific contexts while stabilizing inactive conformations in others, contingent upon cellular redox status and post-translational modifications [31]. Future investigations employing fluorogenic caspase substrates and site-directed mutagenesis will be required to definitively ascertain whether CV functions as a direct caspase agonist or an indirect facilitator of apoptotic signaling.

NF-κB constitutes a master transcriptional regulator of inflammation, survival, and extracellular matrix (ECM) remodeling, with constitutive activation documented in CAFs across multiple carcinoma subtypes [32]. Our immunofluorescence analysis demonstrated elevated cytoplasmic and nuclear p65 (RelA) localization in CAFs relative to NFs (*p* < 0.0001), consistent with pathway hyperactivation. Notably, CV treatment (400 μM, 24 h) significantly attenuated nuclear p65 accumulation in CAFs (*p* < 0.01) while sparing NFs, indicating selective disruption of NF-κB signaling in the activated stromal compartment. This finding is particularly salient given that NF-κB drives the transcription of matrix metalloproteinases (MMPs), pro-inflammatory cytokines, and anti-apoptotic proteins, collectively fostering a permissive niche for tumor invasion and metastasis [33].

Matrix metalloproteinases, notably MMP-1, MMP-2, and MMP-3, are zinc-dependent endopeptidases that degrade ECM components, facilitating cancer cell migration and angiogenesis. Our Western blot analyses revealed context-dependent MMP modulation: MMP-3 was uniformly suppressed in both NFs and CAFs following CV exposure (*p* < 0.01), whereas MMP-2 exhibited a paradoxical increase in CAFs, restoring levels toward the NF baseline (*p* < 0.001). MMP-1, conversely, was not repressed and showed modest induction in NFs. This heterogeneous response underscores the complexity of stromal ECM remodeling and suggests that CV does not impose a global MMP blockade but rather recalibrates specific proteolytic axes. The suppression of MMP-3, an enzyme implicated in collagen degradation and pro-MMP-9 activation, may attenuate invasive potential, whereas the normalization of MMP-2 could reflect compensatory stromal adaptation or feedback regulation [34]. Future studies employing zymography and ECM degradation assays will be essential to validate these biochemical alterations functionally.

Peroxisome proliferator-activated receptors (PPARs) are ligand-activated transcription factors that govern lipid metabolism, mitochondrial biogenesis, and inflammatory responses. PPARα, predominantly expressed in tissues with high fatty acid oxidation capacity, has been implicated in CAF metabolic reprogramming, wherein stromal fibroblasts undergo a catabolic shift to supply lactate, ketone bodies, and amino acids to adjacent cancer cells—a phenomenon termed the “reverse Warburg effect” [27]. Our immunofluorescence and Western blot analyses revealed that PPARα is basally elevated in CAFs relative to NFs (*p* < 0.001) and is selectively downregulated by CV in CAFs (*p* < 0.05) but not in NFs. This CAF-selective suppression suggests that CV disrupts metabolic symbiosis between stroma and epithelium, potentially curtailing the nutrient supply that sustains cancer cell proliferation. Conversely, PPARγ, which has been ascribed both tumor-suppressive and tumor-promoting roles depending on cellular context, remained largely unaffected by CV treatment in our system. This dichotomy is consistent with recent literature indicating that PPARγ in CAFs can drive adipocyte-like differentiation and lipid accumulation, whereas PPARα governs oxidative metabolism and ketogenesis. The selective targeting of PPARα by CV may therefore represent a mechanism to disrupt catabolic reprogramming without perturbing PPARγ-dependent adipogenic differentiation, which in certain contexts can exert anti-tumorigenic effects. Mechanistically, PPARα downregulation may be mediated by CV-induced oxidative stress, post-translational modifications, or transcriptional repression via NF-κB inhibition, given the documented crosstalk between these pathways [35]. These findings resonate with contemporary ‘reverse Warburg’ models proposing that oxidative and ketogenic CAFs fuel epithelial tumor cells via lactate/ketone bodies and fatty acid oxidation, suggesting that CV may function as an agent capable of functionally decoupling this stromal–epithelial metabolic crosstalk [26,36].

Regarding SIRT1 and SIRT3, our Western blot analyses demonstrated that CV selectively elevates SIRT1 and SIRT3 expression in CAFs (*p* < 0.01 and *p* < 0.05, respectively), with minimal effects in NFs. This upregulation may represent a compensatory stress response to CV-induced mitochondrial perturbation, oxidative damage, or NAD^+^ depletion. SIRT3, in particular, governs mitochondrial protein acetylation, antioxidant defense (via SOD2 deacetylation), and apoptotic priming, with overexpression reported to sensitize certain cancer cell types to apoptosis [37]. The concurrent elevation of SIRT1 and SIRT3, alongside increased BAX/BCL-XL ratios, suggests that CV-induced sirtuin activation may paradoxically promote apoptosis in CAFs, potentially by deacetylating pro-apoptotic proteins or by amplifying mitochondrial reactive oxygen species (ROS) production. This scenario aligns with the contemporary ‘double-edged sword’ literature, which emphasizes that SIRT1/SIRT3 can elicit either pro- or anti-apoptotic outcomes depending on cancer type, by simultaneously buffering oxidative stress while recalibrating mitochondrial priming and BCL-2 family signaling [38,39,40].

As for autophagy, Chaperone-mediated autophagy (CMA), a selective degradation pathway mediated by LAMP2A and HSC70, has been shown to exhibit context-dependent pro- or anti-tumorigenic roles [19]. Our data reveal that CV reduces LAMP2A expression in both NFs and CAFs, with a more pronounced decrement in NFs (*p* < 0.01–0.001), while concomitantly inducing p62/SQSTM1 accumulation selectively in CAFs (*p* < 0.01). Notably, LAMP2A-mediated CMA has been shown to promote breast cancer angiogenesis and aerobic glycolysis in MCF-7 cells via HK2-dependent lactate production and VEGFA upregulation, suggesting that CV-induced LAMP2A downregulation may disrupt not only proteostasis but also metabolic reprogramming and tumor vascularization [18]. p62 is an autophagy receptor that targets ubiquitinated cargo for autophagic degradation; its accumulation is a canonical marker of impaired autophagic flux [41]. In our setting, selective p62 accumulation in CAFs, together with LAMP2A down-regulation, suggests that CV may compromise components of macroautophagy and/or CMA in the activated stroma, imposing proteostasis stress; however, definitive flux measurements (e.g., LC3 turnover assays) will be required to establish this directly. Notably, p62 accumulation has been linked to NF-κB activation and oxidative stress, suggesting a feed-forward loop in which autophagic blockade amplifies inflammatory signaling and increases apoptotic susceptibility [42].

Collectively, our findings support a multi-nodal model in which CV selectively reprograms CAFs through coordinated modulation of NF-κB, PPAR, sirtuin, and autophagy networks (Figure 8B). In this framework, CV-induced NF-κB suppression attenuates pro-survival and pro-inflammatory transcriptional programs, while PPARα downregulation disrupts metabolic symbiosis and nutrient provisioning to cancer cells. Concurrently, SIRT1/SIRT3 upregulation and an altered autophagy/CMA marker profile (LAMP2A reduction, p62 accumulation) impose proteostasis stress, and mitochondrial dysfunction, culminating in BAX/BCL-XL-mediated MOMP and caspase-dependent apoptosis. The molecular dynamics simulations provide mechanistic plausibility for direct caspase engagement, although enzymatic validation remains requisite.

This integrative model resonates with emerging paradigms emphasizing the therapeutic potential of stromal reprogramming over stromal ablation. Accumulating experimental evidence demonstrates that CAF heterogeneity encompasses functionally distinct subpopulations with divergent, and occasionally antagonistic, roles in tumor progression. Single-cell transcriptomic profiling of breast cancer models has revealed that tissue-resident normal fibroblasts differentiate into at least two major CAF subtypes—inflammatory CAFs (iCAFs) and myofibroblastic CAFs (myCAFs)—with CD26^+^ normal fibroblasts predisposed to transition into pro-tumorigenic iCAFs that secrete CXCL12, recruit immunosuppressive myeloid cells, and enhance tumor invasion via matrix metalloproteinase activity [43]. Conversely, preclinical investigations have identified tumor-restraining CAF subpopulations, including Meflin^+^ fibroblasts and proto-myofibroblasts exhibiting low α-SMA expression, which restrict cancer cell proliferation, promote differentiation, and deposit collagen matrices that physically constrain tumor expansion [44]. Importantly, indiscriminate CAF depletion strategies have been shown to eliminate these tumor-inhibitory subsets, thereby paradoxically accelerating metastatic dissemination and compromising immune surveillance. Our observation that CV selectively induces apoptosis in activated CAFs—characterized by elevated α-SMA, FAP, PPARα, and constitutive NF-κB signaling—while sparing patient-matched normal fibroblasts suggests a more refined therapeutic paradigm: preferential elimination of pro-tumorigenic CAF subpopulations while preserving quiescent stromal cells and potentially tumor-restraining fibroblast subsets.

From a translational standpoint, the 400 μM concentration used here should be interpreted as an in vitro mechanistic probe dose rather than a clinically extrapolated exposure. Preclinical studies in rodent carcinogenesis models have typically administered carvacrol at 20–100 mg/kg/day, achieving chemopreventive or anti-tumor effects without overt systemic toxicity in colon and breast cancer settings [15,45,46,47,48]. Consistent with these dose ranges, a recent DMBA-induced rat breast cancer model reported that CV at 100 mg/kg given three times per week (oral or intraperitoneal) for 12 weeks significantly reduced tumor burden, activated p53/p73–cytochrome c–TRAIL apoptotic signaling, and improved hepatic and renal function without evidence of intolerable systemic toxicity [49]. Pharmacokinetic investigations further indicate that carvacrol and related monoterpenes undergo rapid first-pass metabolism to glucuronide and sulfate conjugates, with only low-micromolar levels of the free aglycone detectable in plasma and tissues [50]. Thus, while our data demonstrate that breast CAFs are selectively vulnerable to high local concentrations of CV under controlled culture conditions, future in vivo studies will be required to determine whether similar CAF-selective effects can be achieved under pharmacologically realistic dosing regimens and optimized formulations.

### Limitations

Several limitations temper the interpretation of our findings. First, the patient cohort (*n* = 9 CAF cultures, *n* = 5 NF cultures) is modest, and inter-patient heterogeneity in CAF phenotype, genetic background, and prior treatment exposure may influence CV responsiveness. In addition, our CAF cultures were generated from a relatively homogeneous cohort of ER/PR-positive, grade II–III invasive ductal carcinomas, and the present findings are therefore most directly applicable to luminal-type breast cancer; CAF biology in HER2-enriched or triple-negative disease may differ and warrants dedicated investigation. Larger, stratified cohorts with matched clinical metadata (tumor subtype, stage, receptor status) will be essential to identify predictive biomarkers of CV sensitivity. Second, our study employed a single CV concentration (400 μM) and time point (24 h for most assays); dose–response kinetics and time-course analyses across a broader range (e.g., 100–800 μM, 6–72 h) would more comprehensively define the therapeutic window. Third, the molecular dynamics simulations, while informative, are inherently reductionist and do not account for cellular context (e.g., post-translational modifications, cofactor availability, allosteric regulators). Experimental validation through site-directed mutagenesis, co-immunoprecipitation, and enzymatic assays is imperative. Fourth, we did not directly assay caspase-3/-9 activity or cleaved caspase-3/PARP, so caspase involvement in CV-induced apoptosis is strongly suggested by TUNEL/BAX–BCL-XL and MD data but awaits direct biochemical confirmation. Finally, although our CAF and NF cultures are primary cells derived directly from patient tumors, they remain a reductionist ex vivo system that does not capture systemic pharmacokinetics, immune components, or organ-level toxicity; in vivo models will therefore be required to establish the therapeutic index and stromal selectivity of CV more definitively.

## 4. Materials and Methods

**Figure 9:**
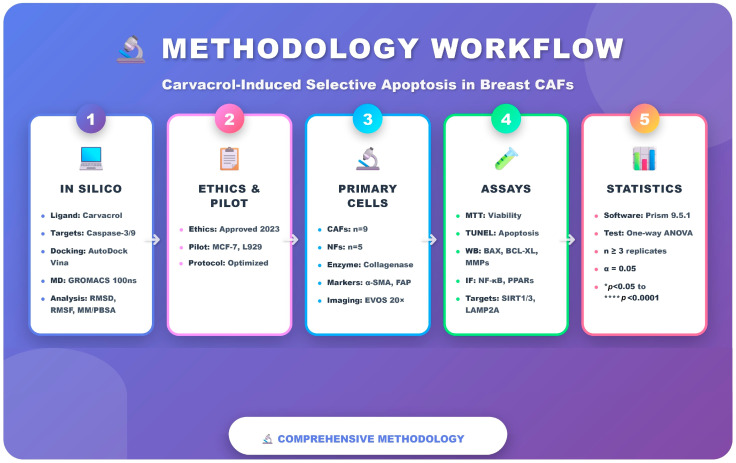
Methodology workflow for carvacrol-induced selective apoptosis in breast CAFs. Schematic overview of the integrated pipeline, including in silico docking and 100-ns MD simulations for caspase-3/9, ethical approval and pilot optimization, isolation and characterization of primary CAFs (*n* = 9) and NFs (*n* = 5), in vitro assays (MTT, TUNEL, Western blot and immunofluorescence for NF-κB, PPARs, SIRT1/3 and LAMP2A), and statistical analysis using one-way ANOVA (GraphPad Prism).

An overview of the integrated methodological workflow used in this study, combining in silico modeling, primary CAF/NF cultures and in vitro assays, is illustrated in Figure 9.

### 4.1. In Silico Methods

#### 4.1.1. Ligand and Target Preparation

Carvacrol (2-methyl-5-isopropylphenol) was downloaded from PubChem (SDF), converted to 3D, and protonated for pH 7.4. Relevant tautomers were enumerated and the dominant microspecies was retained. Geometries were energy-minimized with MMFF94 (aromaticity perceived; ring planarity enforced). Rotatable bonds and the torsion tree were defined following AutoDock—http://autodock.scripps.edu (accessed 30 April 2025) conventions, and the ligand was saved as PDBQT (docking) and MOL2/PDB (MD).

High-resolution human caspase structures (PDB 2AR9 for caspase-3; PDB 5I9B for caspase-9) were retrieved from the Protein Data Bank and pre-processed in UCSF Chimera—https://www.cgl.ucsf.edu/chimera/ (accessed 30 April 2025) [51]. Nonessential heteroatoms and bulk waters were removed, while structurally conserved/bridging waters in the active site were retained if they stabilized pocket geometry. Polar hydrogens were added; alternate conformers were pruned; missing side chains were patched. Each model then underwent stacked minimization in Chimera using the AMBER ff14SB protein force field for standard residues and AM1-BCC (or Gasteiger)—i.e., steepest-descent followed by conjugate-gradient cycles under light positional restraints—to relieve local strain prior to docking and MD. Prepared receptors were exported as PDBQT (docking) and retained as all-atom PDB (MD).

#### 4.1.2. Carvacrol–Caspase Blind Docking

Docking was performed with AutoDock Vina in blind mode using a single grid that encompassed the entire protein (grid box padded to exceed the maximum molecular dimensions by ≥10–12 Å in each axis). Exhaustiveness was increased to enhance global sampling, and multiple independent runs (distinct random seeds) were executed. Resulting poses were clustered at 2.0 Å RMSD; top-scoring clusters were prioritized based on (i) consensus across runs, (ii) internal score rank, and (iii) chemically plausible contacts (H-bonds, π/π-cation, proximity to the catalytic region or canonical subsites). No prior bias to a known pocket was used.

#### 4.1.3. Molecular Dynamics Simulations

Following blind docking (global search over the entire protein surface), the top-scoring pose(s) from the highest-ranked clusters were advanced to all-atom MD to assess binding-mode stability and protein–ligand compatibility. Simulations were executed with GROMACS (https://www.gromacs.org; accessed 30 June 2025) [52] on the SiBioLead LLP platform (https://sibiolead.com/) [53,54]. Complexes were placed in triclinic periodic boxes and solvated with SPC water. The OPLS/AA force field was used for the protein; ligand parameters were derived from AM1-BCC charges (consistent with prior preparation) and converted to the corresponding OPLS/AA format. Systems were neutralized and brought to 150 mM NaCl by adding counterions. Energy minimization employed steepest descent (5000 steps) to remove close contacts. Equilibration proceeded under NVT and then NPT conditions (100 ps each) at 300 K and 1.0 bar. Unless otherwise noted, the velocity-rescale thermostat and Parrinello–Rahman barostat were used; PME handled long-range electrostatics (real-space cutoff 1.0 nm), with a 1.0 nm Lennard-Jones cutoff and a force-switch/smoothing scheme. All bonds to hydrogens were constrained (LINCS), enabling a 2 fs integration timestep. Production MD ran for 100 ns per complex, writing coordinates every 20 ps (≈5000 frames per trajectory). Halo and Apo forms were completed in triplicate (three repeats; *n* = 3) for each condition. Trajectory quality and binding persistence were evaluated by RMSD (protein backbone; ligand heavy atoms), RMSF (per-residue), radius of gyration (Rg), protein–ligand hydrogen bonds (occupancy %; ≤3.5 Å and ≥135°), and solvent-accessible surface area (SASA) for the ligand and catalytic residues. End-point free-energy estimates were computed via MM/PBSA using gmx_MMPBSA [55], sampling evenly spaced snapshots from the production run; per-residue decomposition highlighted hot-spot contributions to binding.

### 4.2. In Vitro Assays

#### 4.2.1. Pilot Cell-Line Culture (MCF-7 and L929)

As an initial feasibility step, human breast adenocarcinoma cells MCF-7 (ATCC^®^, Manassas, VA, USA, HTB-22™; RRID:CVCL_0031) and mouse fibroblasts L929 (ATCC^®^ CCL-1™; RRID:CVCL_0462) were cultured under standard conditions to optimize plating density, attachment, and staining parameters prior to experiments with primary cells. Cells were maintained in DMEM/F-12 + 10% FBS, penicillin 100 U/mL, streptomycin 100 μg/mL, and Normocin 100 μg/mL at 37 °C, 5% CO_2_, with medium changes every 3 days. Monolayers were passaged at ~70–80% confluence using trypsin-EDTA and split 1:3 every 6–7 days. All subsequent fixation, immunofluorescence (IF), and imaging settings were harmonized to these pilot conditions.

#### 4.2.2. Primary Breast Stromal Fibroblast Isolation, Culture, and CAF Characterization

Primary stromal-derived breast fibroblasts were processed immediately upon receipt of the surgical specimen: adipose and necrotic areas were trimmed away, tissues were rinsed 3× in PBS, and finely minced (≈1–2 mm fragments) to shorten the enzymatic incubation. Minced tissue was incubated overnight (12–16 h) at 37 °C with Collagenase/Hyaluronidase (10×; STEMCELL Technologies, Vancouver, BC, Canada, Cat. 07912; used at 1× in serum-free DMEM/F-12) under gentle agitation. After the enzymatic reaction, the slurry was mechanically sheared by repeatedly passing it (20–25 strokes) through the needle of an ejector syringe (18–23 G) to disrupt residual aggregates; the reaction was then quenched with an equal volume of 10% FBS/DMEM-F12. The suspension was clarified through a 70 μm nylon strainer, and the fibroblast-enriched fraction was collected by low-speed centrifugation (200× *g*, 3 min, then 350× *g*, 5 min) for downstream culture. The full workflow is shown in Supplementary File S1 Figure S1. To facilitate initial attachment of delicate primary cells, the plating medium contained 20% (FBS Gibco™, Grand Island, NY, USA, Cat. 10500064) (DMEM/F-12, penicillin 100 U/mL, streptomycin 100 μg/mL, Normocin 100 μg/mL). After firm adhesion (24–72 h, sample-dependent), cultures were continued in 10% FBS under the same conditions as the pilot lines (37 °C, 5% CO_2_; medium refreshed every 3 days). Cells were passaged at ~70–80% confluence with trypsin-EDTA and expanded 1:3 as needed Supplementary File S1 Figure S2.

For CAF immunophenotyping, round coverslips in 24-well plates were seeded at 10,000 cells/well. At ~70–80% confluence, cells were fixed (4% paraformaldehyde, 15 min, RT), permeabilized/blocked, and incubated overnight at 4 °C with primary antibodies against α-SMA (Affbiotech, Cincinnati, OH, USA, #BF9212, 1:200) and FAP (Elabscience, Houston, TX, USA, EAB-32870, 1:100), followed by fluorophore-conjugated secondaries and DAPI counterstain [56]. Images were acquired on an EVOS FL Auto (Thermo Fisher Scientific, Waltham, MA, USA) at 20× (five fields/condition), and CAF abundance was quantified in Fiji/ImageJ (signal per cell), comparing primary CAFs with normal fibroblasts processed in parallel. Isotype/no-primary controls and identical acquisition settings were used across groups. Clinical profiles for all cases are provided in Supplementary File S1 Table S1. For NF cultures, macroscopically normal breast tissue was sampled from areas distant from the invasive front, and these regions were verified as non-malignant by a board-certified pathologist on routine H&E sections before cell isolation. All primary cultures displayed typical fibroblastoid morphology and lacked obvious epithelial-like islands under phase-contrast microscopy during early passages.

#### 4.2.3. MTT Cell-Viability Assay

CAFs and NFs (normal fibroblasts) were seeded in 96-well plates at 3000 cells per well in 100 μL DMEM/F-12 containing 10% FBS and incubated overnight at 37 °C, 5% CO_2_. Carvacrol (CV) was dissolved in DMSO and diluted in culture medium to 0–1500 μM; the final DMSO was ≤0.1% (*v*/*v*) for all conditions. Vehicle controls received matched DMSO without CV. Cells were exposed for 24 h or 48 h. At the end of treatment, 10 μL MTT solution (5 mg/mL) was added to each well and plates were incubated for 3 h at 37 °C. Supernatants were discarded, 100 μL DMSO was added to dissolve formazan, and plates were gently shaken for 5 min. Absorbance was recorded at 570 nm on a microplate reader AMR-100 microplate reader (Allsheng Instruments Co., Ltd., Hangzhou, China) with blank wells for background. Each condition was run in technical triplicates and repeated in ≥3 independent experiments. For downstream assays, CV concentrations and exposure times were chosen below the IC50 (non-toxic range) [57,58].

### 4.3. Western Blotting

To evaluate the effect of carvacrol (CV) on MMP-1, MMP2, MMP-3, Bax, Bcl-xl (BCL2L1), p62 (SQSTM1), Lamp2a, SIRT1, SIRT3, and PPARA, total protein was extracted from cultured cells. Monolayers were rinsed in ice-cold PBS and lysed in Tris lysis buffer (150 mM NaCl, 50 mM Tris, pH 8.0, 1% Triton X-100) supplemented with a 1% protease-inhibitor cocktail. Lysates were clarified by brief centrifugation at 4 °C, and protein concentration (mg/mL) was determined on a NanoDrop DS-11 FX using absorbance at 280 nm (with 260/280 quality ratios recorded). Equal protein amounts were resolved on 4–12% Bis-Tris SDS-PAGE gels (NuPAGE™, NP0322BOX, 1.0 mm; Thermo, Waltham, MA, USA) and transferred to PVDF membranes (ADV-K-12043-C20). Membranes were blocked for 1 h at room temperature in 5% skim milk and then incubated overnight at 4 °C with primary antibodies (all 1:1000): MMP-1 (Elabscience, E-AB-13831), MMP-3 (CST, Danvers, MA, USA, 14351S), MMP-2 (BT-Lab, Wuhan, China, BT-AP05482), SIRT1 (CST, Danvers, MA, USA, 9475S), SIRT3 (CST, Danvers, MA, USA, 5490S), PPARA (Santa Cruz Biotechnology, Dallas, TX, USA, SC-398394), Bax (Novus Biologicals, Centennial, CO, USA, NB120-7977), Bcl-xL (CST, Danvers, MA, USA, 2764), p62 (Elabscience, Wuhan, China, E-AB-62289), Lamp2a (Abcam, Cambridge, UK, Ab18528) and β-actin (CST, Danvers, MA, USA, #3700, 8H10D10). After washes, membranes were probed with HRP-conjugated secondary antibodies appropriate to the primaries—goat anti-mouse HRP (BioLegend, San Diego, CA, USA, 405306) or donkey anti-rabbit HRP (BioLegend, San Diego, CA, USA, 406401). Signals were developed using enhanced chemiluminescence (WesternBright Sirius, K-12043-D10, Advansta, San Jose, CA, USA) and imaged on an iBRIGHT FL1000 (Invitrogen/Thermo Fisher Scientific, Waltham, MA, USA). Band intensities were quantified by densitometry, and each target protein was normalized to β-actin; results are reported as target/β-actin ratios [57,58].

### 4.4. Immunofluorescence Assay

Primary cells were exposed for 24 h to the MTT-nominated, non-toxic carvacrol (CV) doses and then fixed in 4% paraformaldehyde in PBS for 15 min at room temperature (RT). After PBS washes, samples were blocked for 1 h at RT in 1% BSA/PBS. Coverslips were incubated overnight at 4 °C with the following primary antibodies: NF-κB (p65) (1:100, 8242S, Cell Signaling Technology), PPARA (1:50, sc-398,394, Santa Cruz), and PPARG (1:50, sc-7273, Santa Cruz). Following PBS washes, fluorophore-conjugated secondary antibodies were applied for 1 h at RT according to host species: goat anti-rabbit IgG, Alexa Fluor™ 633 (1:400, A-21070, Thermo Fisher, Waltham, MA, USA) or goat anti-mouse IgG, Alexa Fluor™ 488 (1:400, A-11001, Thermo Fisher). Nuclei were counterstained using Fluoroshield Mounting Medium with DAPI (Abcam, AB-104139). Images were acquired on an EVOS^®^ FL Auto (AMF4300, Invitrogen) at 20×, capturing five non-overlapping fields per condition with identical exposure settings. Cell counts and staining intensities were quantified in Fiji/ImageJ (v1.54), and signal was summarized semi-quantitatively using a modified H-score (0–300) [57,58].

### 4.5. TUNEL Assay

Apoptotic DNA fragmentation in primary cultures was assessed using the One-Step TUNEL In Situ Apoptosis Kit (Green/FITC) (Elabscience, E-CK-A320) according to the manufacturer’s instructions, with minor adaptations. Primary cells were plated at 15 × 10^3^ cells per well on sterile glass coverslips in 24-well plates, allowed to attach, and then exposed to the indicated experimental treatments. At the chosen endpoint, culture medium was removed and cells were washed twice with PBS, fixed in 4% paraformaldehyde for 15 min at room temperature, and washed three times with PBS. Permeabilization was performed with 0.2% Triton X-100 for 5 min at room temperature. Samples were equilibrated with 100 μL TdT Equilibration Buffer (37 °C, 20 min), then incubated with 50 μL labeling working solution (FITC-dUTP+TdT in equilibration buffer) for 60 min at 37 °C in the dark. After three PBS washes, coverslips were mounted using DAPI-containing mounting medium. Fluorescence images were acquired on an EVOS^®^ FL Auto (AMF4300, Invitrogen) using FITC and DAPI filter sets at 20× magnification, capturing five non-overlapping fields per condition with identical exposure settings. Images were acquired at ×20 from five non-overlapping fields per coverslip (≥3 coverslips/condition). For each image field, TUNEL-positive nuclei (FITC, green) and total nuclei (DAPI, blue) were counted in Fiji/ImageJ. For each field, the total number of nuclei (DAPI) and TUNEL-positive nuclei were counted, and the percentage of TUNEL-positive cells was calculated as 100 × (TUNEL^+^/total nuclei). For Figure 6, we present a survival index derived from these counts, defined as 100 × [1 − (TUNEL^+^/total nuclei)], such that higher values indicate greater cell survival and lower values reflect increased apoptosis [59].

### 4.6. Statistical Analysis

Each experiment was performed in at least three independent runs. Results are reported as mean ± SD. Group differences were evaluated in GraphPad Prism 9.5.1 (GraphPad Software, San Diego, CA, USA) using one-way ANOVA, with planned comparisons of the carvacrol (CV) treatment groups against the untreated controls. Significance threshold α = 0.05; annotation: ns *p* ≥ 0.05, * *p* < 0.05, ** *p* < 0.01, *** *p* < 0.001, **** *p* < 0.0001.

## 5. Conclusions

In summary, we demonstrate that carvacrol selectively induces mitochondria-related apoptotic signaling in primary breast cancer-associated fibroblasts through convergent modulation of NF-κB, PPARα, SIRT1/SIRT3, and autophagy pathways, while sparing normal fibroblasts. Complementary molecular dynamics simulations support plausible interactions between caspase-3 and caspase-9, although enzymatic validation is required. These findings position carvacrol as a prototype stroma-directed agent warranting further preclinical and translational investigation. Given the emerging recognition that stromal reprogramming—rather than epithelial targeting alone—may be essential to overcome therapeutic resistance and prevent metastatic relapse, natural compounds such as carvacrol offer a pharmacologically tractable, mechanistically pleiotropic, and potentially synergistic adjunct to conventional breast cancer therapeutics. Future studies integrating patient-derived models, in vivo efficacy assessments, and combinatorial regimens will be critical to advance this paradigm from bench to bedside.

## Data Availability

The original contributions presented in this study are included in the article/Supplementary Material. Further inquiries can be directed to the corresponding authors.

## Supplementary Materials

The following supporting information can be downloaded at: https://www.mdpi.com/article/10.3390/ph19010142/s1.

## Abbreviations

The following abbreviations are used in this manuscript:

ANOVA	Analysis of variance
AP-1	Activator protein 1
BAX	Bcl-2-associated X protein
BCL-XL	B-cell lymphoma–extra large
CAF	Cancer-associated fibroblast
CMA	Chaperone-mediated autophagy
CV	Carvacrol
DCIS	Ductal carcinoma in situ
DMEM	Dulbecco’s modified Eagle medium
DMSO	Dimethyl sulfoxide
ECM	Extracellular matrix
ER	Estrogen receptor
FAP	Fibroblast activation protein
FBS	Fetal bovine serum
IF	Immunofluorescence
ILC	Invasive lobular carcinoma
IDC	Invasive ductal carcinoma
MD	Molecular dynamics
MMP	Matrix metalloproteinase
MTT	3-(4,5-dimethylthiazol-2-yl)-2,5-diphenyltetrazolium bromide
NF	Normal fibroblast
NF-κB	Nuclear factor kappa B
NPT	Constant number, pressure, temperature ensemble
NVT	Constant number, volume, temperature ensemble
OPLS-AA	Optimized potentials for liquid simulations–all atom
PBS	Phosphate-buffered saline
PDB	Protein Data Bank
PPAR	Peroxisome proliferator-activated receptor
PPARα	Peroxisome proliferator-activated receptor alpha
PPARγ	Peroxisome proliferator-activated receptor gamma
PR	Progesterone receptor
RMSD	Root-mean-square deviation
RMSF	Root-mean-square fluctuation
ROS	Reactive oxygen species
SASA	Solvent-accessible surface area
SD	Standard deviation
SEM	Standard error of the mean
SIRT	Sirtuin
SIRT1	Sirtuin 1
SIRT3	Sirtuin 3
SQSTM1	Sequestosome 1
TME	Tumor microenvironment
TUNEL	Terminal deoxynucleotidyl transferase dUTP nick end labeling
