# Supplementary material for: Carvacrol Selectively Induces Mitochondria-Related Apoptotic Signaling in Primary Breast Cancer-Associated Fibroblasts
Source: Pharmaceuticals (Basel). 2026 Jan 14;19(1):142. doi: 10.3390/ph19010142 (PMC12844703; doi:10.3390/ph19010142)
Supplement: Supplementary file 1 [file pharmaceuticals-19-00142-s001.zip › Supplementary File S1.pdf]

Patient cohort and tissue procurement

Breast tissue was collected from 9 patients with invasive ductal carcinoma (IDC) undergoing mastectomy. Where available, patient-matched adjacent normal breast tissue was obtained from macroscopically healthy areas >3–5 cm from the tumor, yielding 6 normal counterparts. All specimens were transported under cold-chain conditions on wet ice in HBSS containing penicillin (100 U/mL) and streptomycin (100 µg/mL) and processed immediately according to the workflow shown in Supplementary Fig. S3. Clinical and pathologic characteristics for each case (sex, operation type, tumor side, size, histopathology, grade, ER/PR status, Ki-67 index, E-cadherin, and availability of matched normal tissue) are provided in Supplementary Table S1.

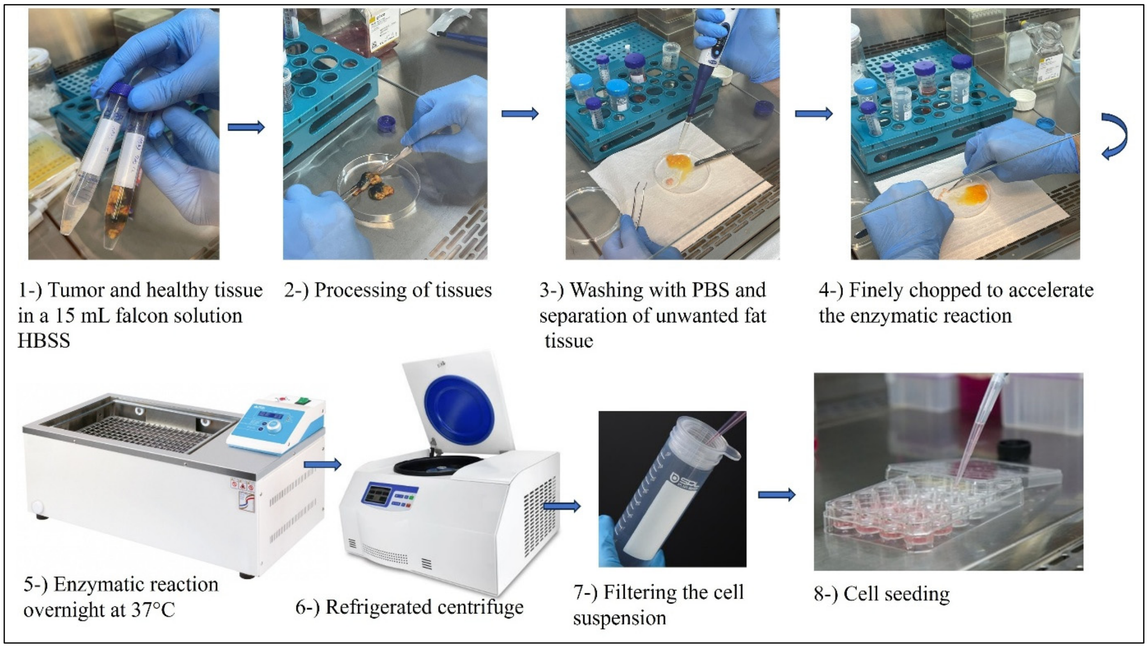

Supplementary Fig. S1 — Primary CAF isolation & culture workflow. Representative montage of the isolation pipeline from fresh surgical tissue: (1) receipt of tumor/healthy tissue in HBSS, (2) gross trimming and removal of visible fat, (3) PBS washes, (4) fine mincing to accelerate enzyme access, (5) overnight enzymatic digestion at 37 °C with Collagenase/Hyaluronidase, (6) low-temperature centrifugation, (7) 70-µm nylon filtering to enrich single cells/fibroblasts, and (8) seeding into 24-well plates. Medium: DMEM/F-12 supplemented as described in Methods; initial FBS was temporarily raised (up to 20%) for primary attachment, then reduced for routine culture. Scale bars not applicable to panels 1–6; seeding images show the indicated scale bars.

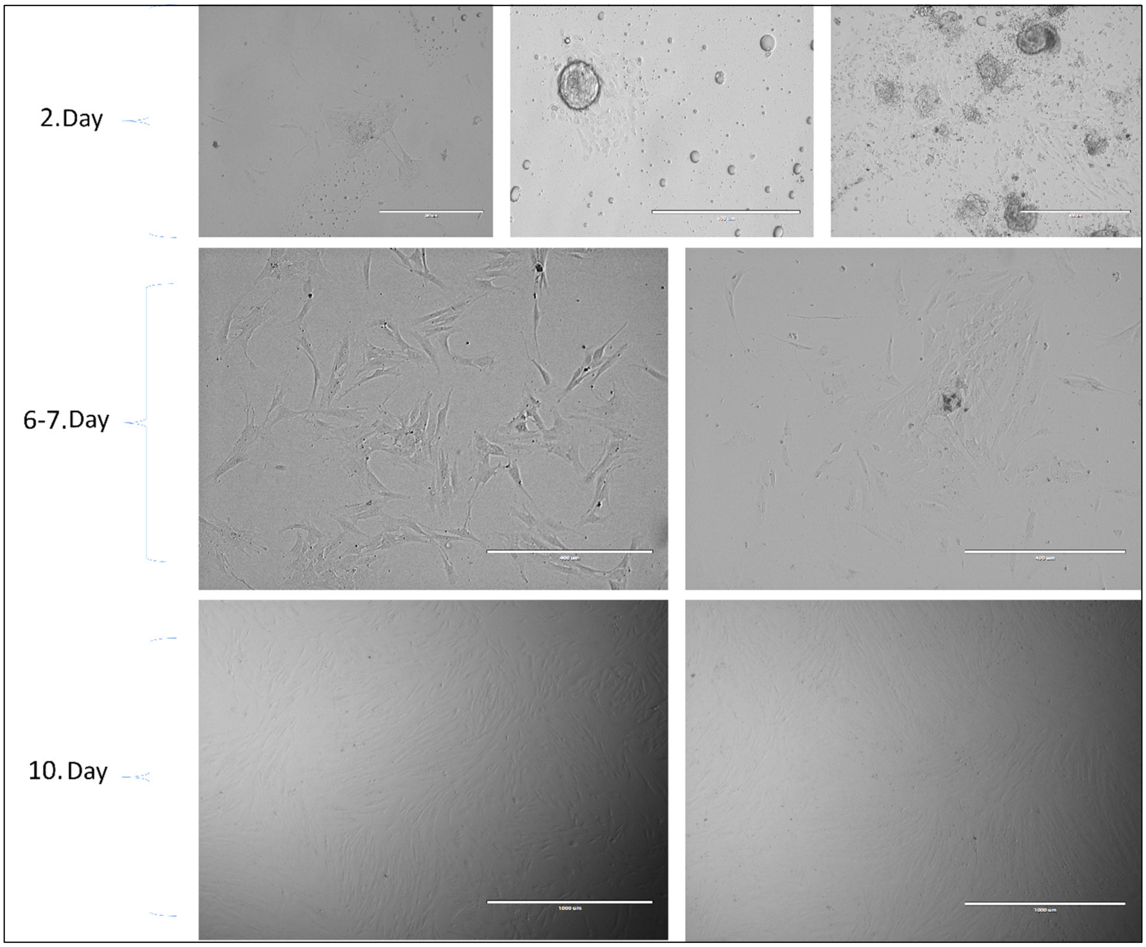

Supplementary Fig. S2 — Expansion of primary CAFs after seeding (phase-contrast). Representative fields at Day 2**,** Day 6–7**,** and Day 10 post-seeding show progressive outgrowth of spindle-shaped fibroblasts and increasing confluence. Early cultures contain occasional tissue fragments that resolve into adherent fibroblast monolayers by Day 6–7; near-confluent sheets are visible by Day 10. Scale bars as indicated on each panel (Day 2/6–7 typically 400 µm; Day 10, 1000 µm).

Supplementary Table S1. Clinicopathologic characteristics of the IDC cohort and availability of matched adjacent normal breast tissue (>3–5 cm from tumor).

	**Patient I**	**Patient II**	**Patient III**	**Patient IV**	**Patient V**	**Patient VI**	**Patient VII**	**Patient VIII**	**Patient XI**	**Patient X**
**Sex**	Female	Female	Female	Female	Female	Female	Female	Female	Female	Female
**Operation type**	Mastectomy	Mastectomy	Mastectomy	Mastectomy	Mastectomy	Mastectomy	Mastectomy	Mastectomy	Mastectomy	Mastectomy
**Tumor side**	Left	Left	Left	Right	Right	Left	Left	Left	Right	Left

**Tumor size (cm)**	1,8x1,5x1,2	2,2x1,8x1,5	2x1,5x2	2x1,7x2,2	2,1x0,8	2,5x2x1,8	2x0,8x0,5	1,1	5,5x2	3,5x3x2
**Pathology**	Malignant	Malignant	Malignant	Malignant	Malignant	Malignant	Malignant	Malignant	Malignant	Malignant
**Histopathological diagnosis**	IDC	IDC	IDC	IDC	IDC	IDC	IDC	IDC	IDC	IDC
**Histological grade**	II	II	II	II	II	II	II	III	II	III
**ER status**	Positive	Positive	Positive	Positive	Positive	Positive	Positive	Negative	Positive	Positive
**PR status**	Positive	Positive	Positive	Positive	Positive	Positive	Positive	Negative	Positive	Positive
**Ki-67 (%)**	30	30	25	45	3-4	10	10	8-10	5	12
**E-cadherin**	Positive	Positive	Positive	Positive	Positive	Positive	Positive	Positive	Positive	Positive
**Matched adjacent normal tissue available**		✓		✓		✓	✓		✓	✓
**Cancer**	✓	✓	✓	✓	✓	✓	✓	✓	✓	

IDC: Invasive Ductal Carcinoma, ER: Estrogen, PR: Progesterone, ✓ Healthy or cancer tissue was obtained from marked patients.

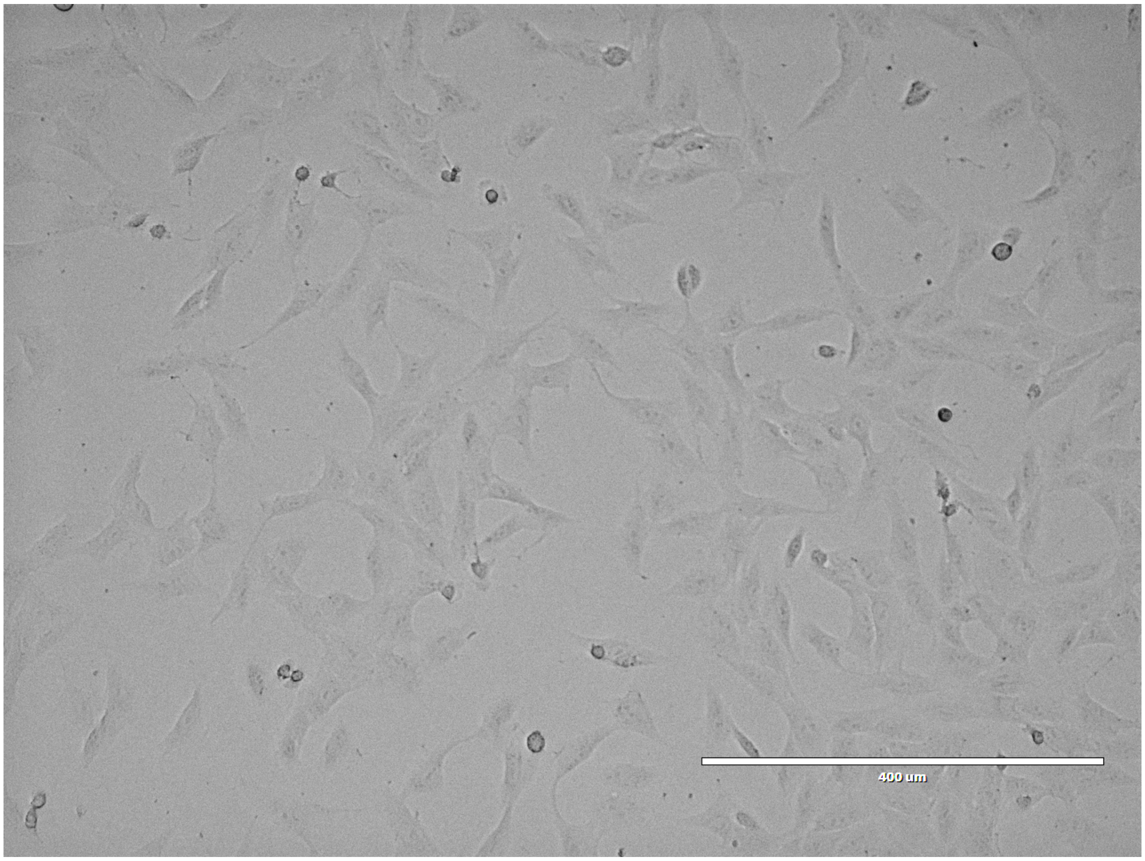

Supplementary Fig. S3. L929 fibroblast morphology**.** Representative micrograph showing spindle-shaped, elongated L929 fibroblasts at ~60–70% confluence with thin lamellipodial/filopodial extensions consistent with a fibroblast phenotype. A few rounded cells are visible (likely mitotic or recently detached). Scale bar**:** 400 µm**.** Image acquired on an inverted phase-contrast microscope (e.g., EVOS FL Auto), 10× objective.

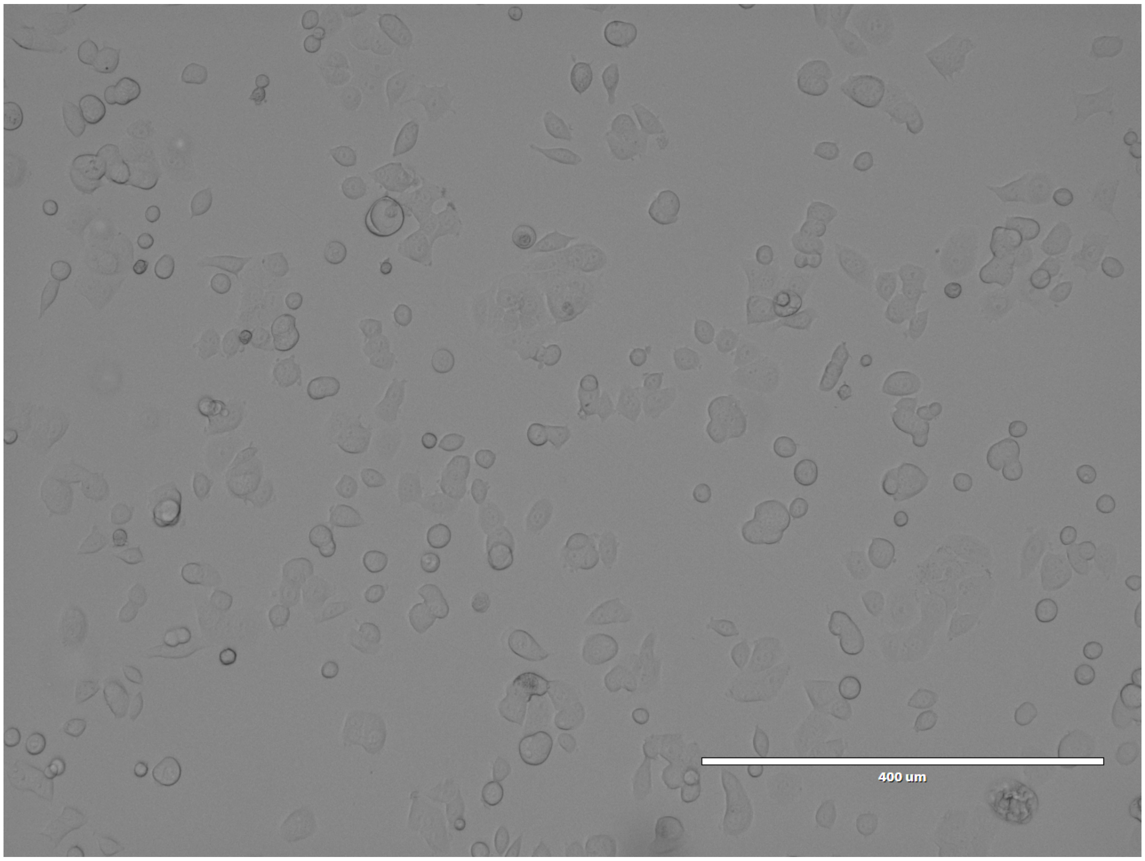

Supplementary Fig. S4 Representative field of MCF-7 human breast adenocarcinoma cells showing the expected polygonal, epithelioid "cobblestone" appearance with tight cell–cell contacts. Scale bar: 400 µm. Image acquired on an inverted phase-contrast microscope (e.g., EVOS FL Auto), 10× objective.

L929 (mouse fibroblast, ATCC CCL-1) and MCF-7 (human breast adenocarcinoma, ATCC HTB-22) were cultured in DMEM (high glucose) + 10% FBS and 1% penicillin–streptomycin at 37 °C/5% CO_2, passaged at ~80% confluence with 0.25% trypsin-EDTA (1:3 split), and imaged at ~70% confluence using EVOS® FL Auto Imaging (AMF4300, Invitrogen) (scale bar 400 µm).
