# Supplementary material for: Carvacrol Selectively Induces Mitochondria-Related Apoptotic Signaling in Primary Breast Cancer-Associated Fibroblasts
Source: Pharmaceuticals (Basel). 2026 Jan 14;19(1):142. doi: 10.3390/ph19010142 (PMC12844703; doi:10.3390/ph19010142)
Supplement: Supplementary file 1 [file pharmaceuticals-19-00142-s001.zip › Supplementary File S2.pdf]

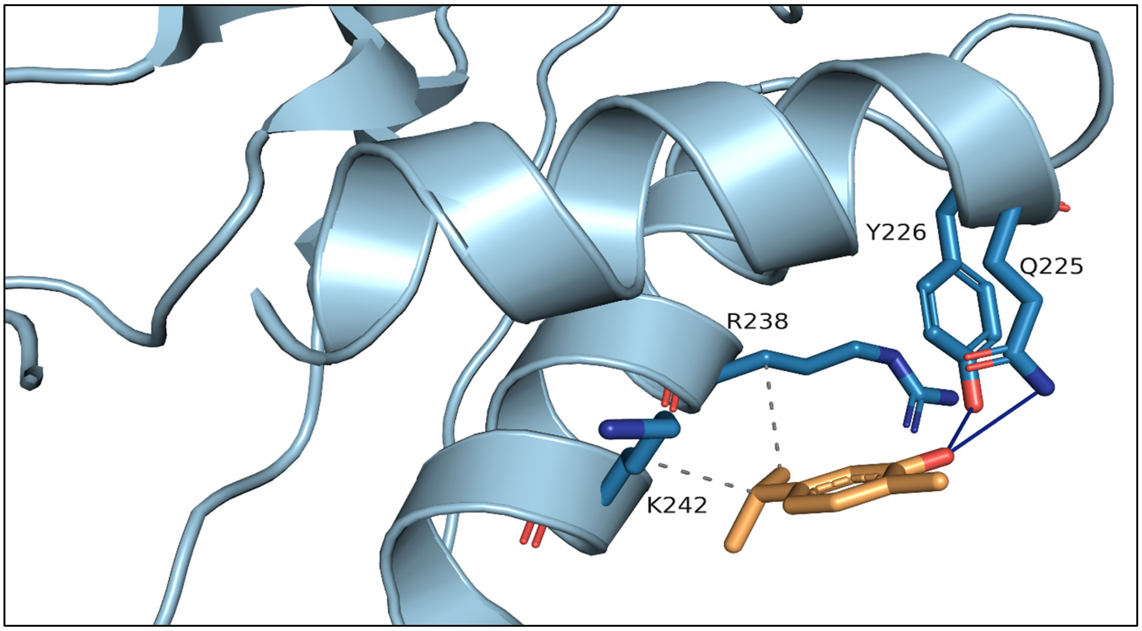

Supplementary Fig. S1 Carvacrol docked to human caspase-3 (PDB: 5I9B). Blind docking over the entire protein surface identified a best-scoring pose for carvacrol (orange sticks; Vina score –5.4 kcal mol^{-1}) in a groove adjacent to the substrate-binding cleft. The phenolic OH engages Gln225 and Tyr226 via hydrogen bonds (solid blue lines), while the aromatic ring forms favorable polar/π-cation contacts with Arg238 and Lys242 (gray dashed lines). Protein is shown as a cartoon; interacting side chains are in sticks and labeled. This top pose was forwarded to MD for stability assessment (see Methods).

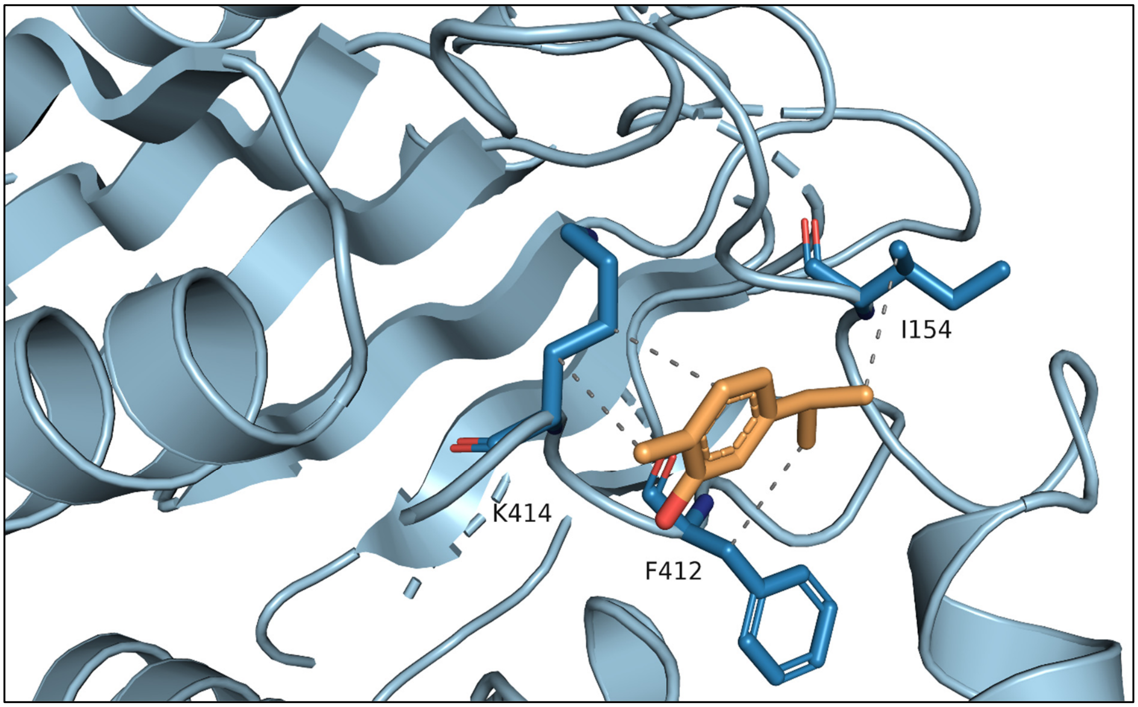

Supplementary Fig. S2 Carvacrol docked to human caspase-9 (PDB: 2AR9). Blind docking located a best-scoring pose for carvacrol (orange sticks; Vina –5.2 kcal·mol^{-1}) in a shallow pocket. The interaction network is purely hydrophobic/π–alkyl—predominantly with Ile154, Phe412, and the aliphatic segment of Lys414 (gray dashed contacts). No hydrogen bonds are present in this pose.

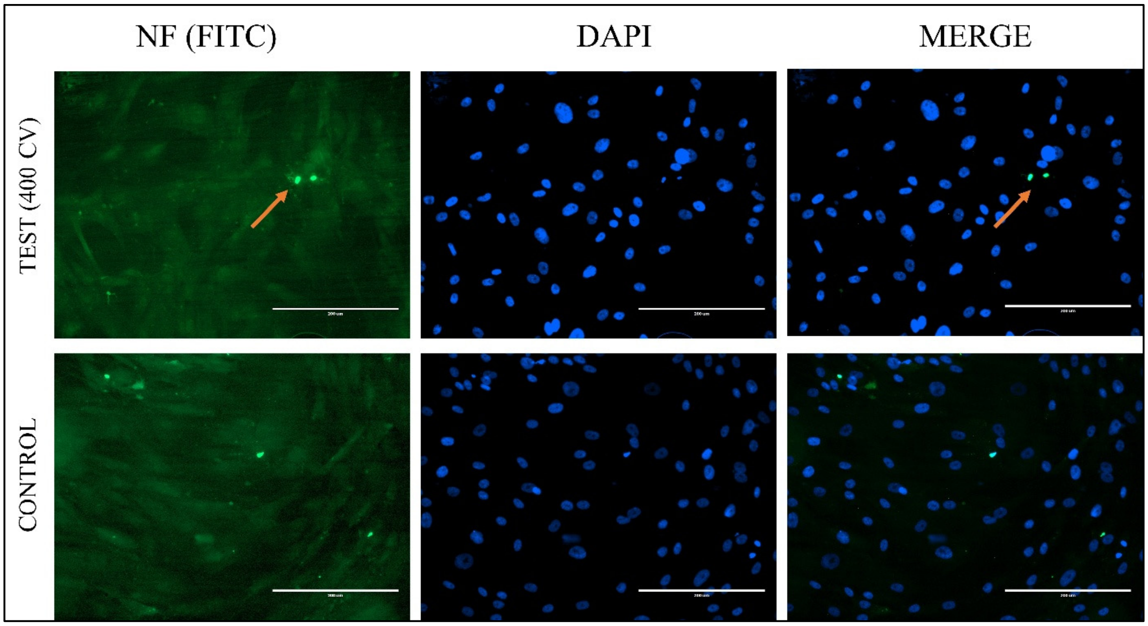

Supplementary Fig. S3. Normal fibroblasts show negligible apoptosis with 400 µM CV. TUNEL IF (FITC/DAPI/merge) for NF cultures ± CV at matched time points. Rare TUNEL-positive nuclei are indicated. Quantification shows **no significant change** in % Surviving Cells or BAX/BCL-XL (n = 3).
